# Using a coloring activity to identify children’s development of visual–motor integration: an application of artificial intelligence

**DOI:** 10.1080/07853890.2025.2578725

**Published:** 2025-11-03

**Authors:** Tzu-Yun Huang, Kuan-Lin Chen, Gong-Hong Lin, Chien-Yu Huang

**Affiliations:** a Department of Occupational Therapy, College of Medicine, National Cheng Kung University, Tainan City, Taiwan; b Institute of Allied Health Sciences, College of Medicine, National Cheng Kung University, Tainan, Taiwan; c Department of Physical Medicine and Rehabilitation, National Cheng Kung University Hospital, College of Medicine, National Cheng Kung University, Tainan City, Taiwan; d International Ph.D. Program in Gerontology and Long-Term Care, College of Nursing, Taipei Medical University, Taipei, Taiwan; e School of Occupational Therapy, College of Medicine, National Taiwan University, Taipei, Taiwan; f Department of Physical Medicine and Rehabilitation, National Taiwan University Hospital, Taipei, Taiwan

**Keywords:** Artificial intelligence, visual–motor integration, coloring

## Abstract

**Aim:**

Visual–motor integration (VMI) is an important indicator in children with learning disabilities. We aimed to use performance in a coloring activity to identify children’s VMI developmental status.

**Methods:**

A sample of 505 preschool children (mean = 57.64, SD = 11.10) were recruited. Among them, data from 404 and 101 children were used as the training and testing data, respectively. The Beery–Buktenica Developmental Test of Visual–motor Integration, fourth Edition, (VMI-4) was used as an indicator for the model of artificial intelligence (AI). The total scores of the VMI-4 were calculated, and then based on the children’s age, the total scores were transferred into standard scores and the developmental status of visual–motor integration. The AI model comprised a regression model and classification model to predict the developmental status rated by the VMI-4.

**Results:**

In the training data, we found that the AI model comprising the support vector machine (SVM) regression model and eXtreme Gradient Boostin (XGBoost) classification model exhibited the best performance (accuracy: 86.2%; sensitivity: 84.7%; and specificity, 85.4%). The results of the trained AI model on the testing data indicated good performance, with accuracy, sensitivity, and specificity of 80.20%, 73.68%, and 81.71%, respectively.

**Conclusions:**

Combining the coloring activity with the AI technique has great potential as a screening tool to identify children’s VMI developmental status.

## Introduction

Coloring is an essential and highly popular activity among preschool children, as it naturally follows their initial scribbling phase. The inherent appeal of colors makes the activity enjoyable and engaging, motivating children to participate independently [1]. Moreover, coloring activities require children to manage boundaries and control the pen or crayon in various directions (e.g. side-to-side, up-and-down, circular). This fosters the development of crucial skills, including visual–motor integration, which is vital for handwriting and academic success [2]. Consequently, educators and clinicians often observe children’s coloring performance to infer their developmental progress. This highlights the potential for coloring activities to serve as a practical medium for identifying children’s developmental status.

Visual–motor integration involves coordinating visual information with the movements of the upper and lower extremities [3]. Early identification of visual–motor integration skills is crucial for preventing learning difficulties in children. During coloring activities, children must control their hand movements while attending to the boundaries of shapes. This process requires eye–hand coordination, making coloring a promising tool for assessing visual–motor integration. For instance, Abou-El-Saad et al. included coloring as a subscale in the assessment of visual–motor integration [4], and Sanghavi and Kelkar [3] demonstrated that children with learning disabilities and poor visual–motor integration struggled with activities such as drawing, cutting, copying designs, and coloring. Huang [5] investigated the associations between the product of the coloring activity and visual–motor integration and found moderate associations between the activity and VMI. This indicates that the quality of a child’s work in coloring activities has high potential to serve as a window into their visual–motor integration abilities.

However, despite the utility of coloring activities in developmental assessments, two significant issues remain unaddressed. First, evaluation framework for assessing performance in coloring activities have limitations. The criteria often depend on the subjective perspectives, background knowledge, and experiences of evaluators, leading to inconsistent judgments. For instance, clinicians might note that a child exhibits poor visual–motor integration based on their inability to stay within boundaries, but such observations are descriptive and subjective, limiting their use for quantitative analysis. This lack of standardization restricts the ability to monitor developmental progress objectively and provide evidence-based assessments. Second, the absence of quantitative tools limits the broader application of coloring activities in assessments. While subjective evaluations offer individualized insights, they cannot be effectively scaled or analyzed for patterns, making it challenging to establish benchmarks or monitor progress over time. Ideally, since coloring activities can serve as a regular tool for monitoring children’s developmental status, they should be accompanied by quantitative measures that complement subjective insights.

Emerging technologies, particularly computer vision and data prediction techniques powered by artificial intelligence (AI), hold great promise for addressing these challenges. Computer vision can transform colored pictures into numerical data, capturing key features of a child’s performance, such as precision, boundary adherence, and color distribution. These numerical data can then be analyzed using predictive algorithms to evaluate visual–motor integration against standardized benchmarks. This approach allows for objective, reproducible, and quantitative assessments of coloring activities. AI methods have already demonstrated success in related applications across medical, rehabilitation, and educational domains. For example, Son [6] utilized virtual reality (VR) to observe and classify actions, diagnosing attention deficit hyperactivity disorder (ADHD) with 98.3% accuracy using convolutional neural networks. Similarly, Ihlen [7] developed an AI model that identified cerebral palsy early by analyzing infant video recordings, achieving high sensitivity (92.7%) and specificity (81.6%). These examples underscore the potential of AI-driven methodologies to deliver precise and reliable developmental assessments. Given this context, the integration of computer vision and data prediction techniques offers a novel pathway to enhance the utility of coloring activities as a tool for assessing visual–motor integration. By enabling standardized, objective, and quantitative evaluations, these technologies can provide valuable insights into children’s developmental status, ultimately improving early identification and intervention strategies.

Integrating coloring activities with AI techniques to assess children’s developmental status in visual–motor integration could have two benefits. First, selecting a coloring activity as the medium allows for exploring whether play-based outcomes can effectively reflect children’s developmental abilities. If children’s developmental status can be identified through their play products, this approach may provide a more accurate representation of their capabilities compared to standardized assessments. Moreover, as coloring is a common and engaging activity, this method could be easily adopted by caregivers, schoolteachers, and clinicians. Second, this approach offers broad accessibility, particularly benefiting regions with limited access to professional practitioners, such as rural areas. Were the AI model incorporated into a webpage or mobile app, teachers and parents could upload children’s coloring images to a cloud platform and receive immediate feedback on their developmental status. For example, Chun [8] employed a similar AI-driven method to assess children’s gross motor behaviors. They developed an automated, labor-free digital system to address challenges posed by limited medical resources. Their findings demonstrated acceptable to good performance, with areas under the receiver operating characteristic curve (AUROCs) ranging from 0.79 to 0.90. This underscores the potential of machine learning approaches for screening and identifying children’s developmental status.

In this context, the aim of our study is to investigate whether children’s performance in coloring activities can effectively identify their visual–motor integration developmental status, leveraging computer vision and AI-based data prediction techniques.

## Methods

### Participants

Preschool children were recruited for this study. The inclusion criteria were (1) age of 3 to 6 years, (2) ability to participate in group activities, and (3) informed consent provided by caregivers. This study was conducted in accordance with the Declaration of Helsinki and its subsequent amendments, and was approved by the Institutional Review Board of E-DA hospital in Kaohsiung, Taiwan [EMRP46107N]. This study was conducted during 2020 to 2021.

### Measures

#### A coloring picture of a train

A picture of a train was used for the coloring activity. Children were asked to color within the boundaries and instructed not to draw other things on the paper.

## Beery–Buktenica developmental test of visual–motor integration, fourth edition (VMI-4)

The VMI-4 was used to assess visual–motor integration in the children [9]. The VMI-4 contains 27 items. Unlike the Beery VMI-6, the Beery VMI-4 does not include the first three items designed for 2-year-old children. During the assessment, children are required to copy geometric forms. Each item is scored as 1 (correct) or 0 (incorrect), with a total score ranging from 0 to 27. The total score is transformed into a standard score. The standard score is further used to identify the developmental status of visual–motor integration as typical development or developmental delay. The total VMI-4 score and developmental status were used in our study. The VMI-4 has good psychometric properties [9].

### Procedures

Prior to formal scoring, the researchers completed a rigorous training process, and a pilot test was conducted to identify and discuss rating discrepancies, ensuring consensus among the researchers. The principals of kindergartens were contacted to recruit participants. Principals and teachers helped distribute letters with research instructions and informed consent forms to the caregivers. Caregivers gave written informed consent to researchers if they agreed to participate in the study. Children whose caregivers agreed to participate in the study then participated in the group activities. The researchers led small groups of 3 to 5 children. During each session, the children completed three activities (origami, coloring, and copying activities) and the VMI-4. Group activities and assessments took 30 to 40 min. The blank train was printed on half of an A4 sheet of paper. Children were instructed to color the train only, without adding any additional drawings. After the group activity, the researchers independently rated the VMI-4. The colored trains were then scanned with a scanner and saved as PDF files. This ensured that the size and resolution of every image remained consistent, minimizing unnecessary variations. Once the coloring pictures and VMI-4 scores were collected, the first author conducted AI prediction analyses. Both the researchers and the first author were blinded to the AI predictions due to the large dataset and the unknown mechanisms by which the AI assessed the children’s developmental status.

### Statistical analyses

The data analysis procedure comprised two steps: data preprocessing and data prediction. Data preprocessing

First, we converted the coloring pictures into quantitative data using EfficientNetb7 [10]. EfficientNetb7, part of the EfficientNet family, which emphasizes scalability and efficiency, is a convolutional neural network architecture originally designed for image classification tasks. It is widely used in computer vision tasks for image classification, object detection and segmentation. Specifically, EfficientNetb7 can utilize the layers of a convolutional neural network to amplify, shrink, filter, concentrate, or apply linear and nonlinear transformations to the pixel values in an image. This process helps extract important numerical information from the image. Considering its ability to deal with images, we applied this model to extract the quantitative features of the images and further used those features for data prediction. By default, EfficientNetB7 generates 2,560 feature variables. While these variables can be treated as a unified representation, their individual meanings are not directly interpretable. EfficientNetB7 has been successfully applied to classification in numerous studies, indicating that 2,560 features can be effectively used for accurate prediction or classification.

Then we split our data by a ratio of 4:1 into the training and testing datasets, which were stratified by children’s developmental status on the VMI-6. Finally, to prevent an imbalance in data between typical development and developmental delay, the synthetic minority oversampling technique (SMOTE) was applied to the training dataset [11]. SMOTE is a popular technique used in machine learning to deal with imbalanced datasets when the data in one category are significantly fewer than those in the other category. Such an imbalance usually causes poor model performance because the machine learning will be biased toward the majority category. SMOTE helps to solve this challenge by generating synthetic data in the minority category. We expected that the proportion of children with typical development would be much greater than that with developmental delay (i.e. imbalanced data) because the data were collected from kindergartens. Imbalanced data might cause AI models to misclassify children as having typical development. Therefore, we used SMOTE to simulate real data on developmental delay so that the ratio of data on typical development to that on developmental delay in the training dataset would be 1:1. Notably, the SMOTE simulation used real data on developmental delay, and it was used only in the training dataset not in the testing dataset. Therefore, it would not influence the final validation of model performance.

#### Data prediction

We explored three approaches to train machine learning models to mimic the process of using the VMI-4 to determine developmental status. In the human rating process, VMI-4 total scores were first evaluated, followed by the determination of developmental status based on VMI-4 norms and the child’s age. To replicate this process, we developed the following approaches:

Approach 1: Applying a machine learning model to analyze images and directly predict VMI-4 total scores, then manually transforming the scores with the child’s age into developmental status.

Approach 2: Applying a machine learning model to both images and the child’s age to predict VMI-4 total scores, followed by manual transformation into developmental status.

Approach 3: Applying a machine learning model to images to first predict VMI-4 total scores and then applying a machine learning model again to the predicted scores and the child’s age to predict developmental status.

For Approaches 1 and 2, we employed a machine learning regressor, while Approach 3 utilized both a regressor and a classifier. The regressor, based on regression algorithms, predicted VMI-4 total scores from quantitative image data. The classifier, based on classification algorithms, predicted developmental status using the predicted VMI-4 total scores and the child’s age.

To optimize model performance, we tested four candidate algorithms for both the regressor and the classifier, tuning their parameters using RandomizedSearchCV. The algorithms included eXtreme Gradient Boosting (XGBoost), Categorical Boosting (CatBoost), Random Forest (RF), and Support Vector Machine (SVM), which were selected for their strong predictive power in health data applications [12–15]. Specifically, we developed four types of regressors for Approaches 1 and 2, and 16 regressor-classifier combinations for Approach 3.

We ensured the reliability of the tuned parameters through 5-fold cross-validation, with performance metrics averaged across five different training–testing splits. The final trained models were applied to the original training dataset (without SMOTE) to test if overfitting of the model existed. Model performance was evaluated using four key indicators: R^2^, sensitivity, specificity, and F1 score. Additionally, Youden’s J statistic (J = sensitivity + specificity − 1) was used to assess diagnostic performance, with *J* > 0.5, 0.7, and 0.9 indicating acceptable, moderate, and high diagnostic power, respectively [16,17].

Finally, we validated the model’s performance using the testing dataset.

## Results

### Characteristics of participants

A total of 505 children were included in this study. Demographic characteristics of the children are presented in Table 1. The data of 404 children were used as the training dataset, while the remaining data were used as the testing dataset. There were no significant differences in age or VMI-4 total scores between the training and testing datasets. Because the numbers of children classified as having typical development (*n* = 408) and developmental delay (*n* = 97) were imbalanced, SMOTE was then applied to the training dataset. The application of SMOTE increased the training dataset to 652 children, and the numbers of children with typical development and developmental delay were even (both *n* = 326) in the training dataset.

**Table 1.** Demographic characteristics of children before using SMOTE (*N* = 505).

	Training dataset (*n* = 404)	Testing dataset (*n* = 101)	*p* value
Gender (male): n (%)	208 (51.49)	48 (47.52)	.476
Biological ages (months): mean (SD)	57.61 (10.82)	57.78 (12.16)	.897
VMI total scores: mean (SD)	14.21 (3.72)	14.65 (3.97)	.290
Developmental status of visual–motor integration (developmental delay): n (%)	78 (19.31)	19 (18.81)	.910

### Performance of AI models

**Figure 1.**
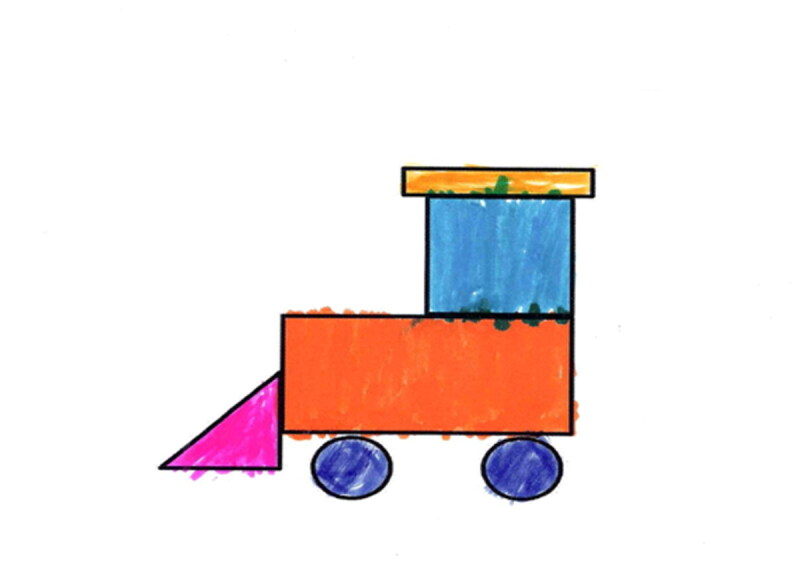
An example of a completed coloring task.

Figure 1 presents an example of a completed coloring task. For Approach 1, the four types of regressors demonstrated suboptimal performance, with R^2^ values only ranging from 0.58 to 0.65. Due to the limited accuracy in predicting VMI-4 total scores, we did not proceed with transforming these scores into developmental status or evaluating further metrics such as sensitivity and specificity.

For Approach 2, Table 2 presents the model performance on the testing dataset. The R^2^ values were moderate, ranging from 0.61 to 0.71, and the specificity was high, ranging from 0.74 to 0.83. However, the sensitivity was relatively low, only ranging from 0.47 to 0.63.

**Table 2.** The model performance using coloring images and age to predict VMI scores and manual transformation into developmental status.

Metrics	XGBoost Regressor	CatBoost regressor	RF Regressor	SVM Regressor
R^2^	0.698	0.708	0.615	0.679
Sensitivity	0.632	0.526	0.474	0.474
Specificity	0.817	0.829	0.744	0.805
F1 score	0.796	0.782	0.716	0.755

*Abbreviations: XGBoost, extreme Gradient Boosting; CatBoost, Categorical Boosting; RF, Random Forest; SVM, Support Vector Machine.

Table 3 shows the performance of Approach 3 for both the original training dataset (without SMOTE) and the testing dataset. The results indicated that the combination of the SVM regressor and XGBoost classifier achieved the best classification performance, with a sensitivity of 1.00, specificity of 0.75, F1-score of 0.82, and AUC of 0.972. The Youden’s J statistic was 0.75, indicating moderate diagnostic power.

**Table 3.** Performance of the 16 combinations with the original training dataset (without SMOTE).

Machine learning model combinations		Training dataset				Testing dataset			
Regressor	Classifier	Sensitivity	Specificity	F1 score	AUC	Sensitivity	Specificity	F1 score	AUC
XGBoost Regressor	XGBoost Classifier	0.974	0.690	0.772	0.926	0.579	0.732	0.729	0.803
	CatBoost Classifier	0.872	0.899	0.898	0.943	0.368	0.890	0.785	0.808
	RF Classifier	0.872	0.896	0.896	0.928	0.368	0.878	0.777	0.798
	SVM Classifier	0.795	0.936	0.909	0.921	0.316	0.927	0.794	0.509
CatBoost Regressor	XGBoost Classifier	1.00	0.721	0.798	0.956	0.684	0.720	0.741	0.788
	CatBoost Classifier	1.00	0.963	0.971	0.995	0.474	0.915	0.826	0.743
	RF Classifier	1.00	0.963	0.971	0.993	0.474	0.902	0.818	0.876
	SVM Classifier	1.00	0.991	0.993	0.998	0.368	0.915	0.800	0.607
RF Regressor	XGBoost Classifier	1.00	0.693	0.778	0.966	0.789	0.634	0.700	0.794
	CatBoost Classifier	0.974	0.954	0.959	0.978	0.474	0.805	0.755	0.688
	RF Classifier	0.949	0.936	0.940	0.967	0.526	0.829	0.782	0.817
	SVM Classifier	0.923	0.963	0.956	0.973	0.421	0.890	0.798	0.634
**SVM Regressor**	**XGBoost Classifier**	**1.00**	**0.748**	**0.817**	**0.972**	**0.737**	**0.805**	**0.808**	**0.829**
	CatBoost Classifier	0.987	0.945	0.955	0.974	0.421	0.902	0.806	0.732
	RF Classifier	0.987	0.933	0.945	0.974	0.421	0.902	0.806	0.746
	SVM Classifier	0.949	0.969	0.966	0.978	0.368	0.915	0.800	0.493

**Figure 2.**
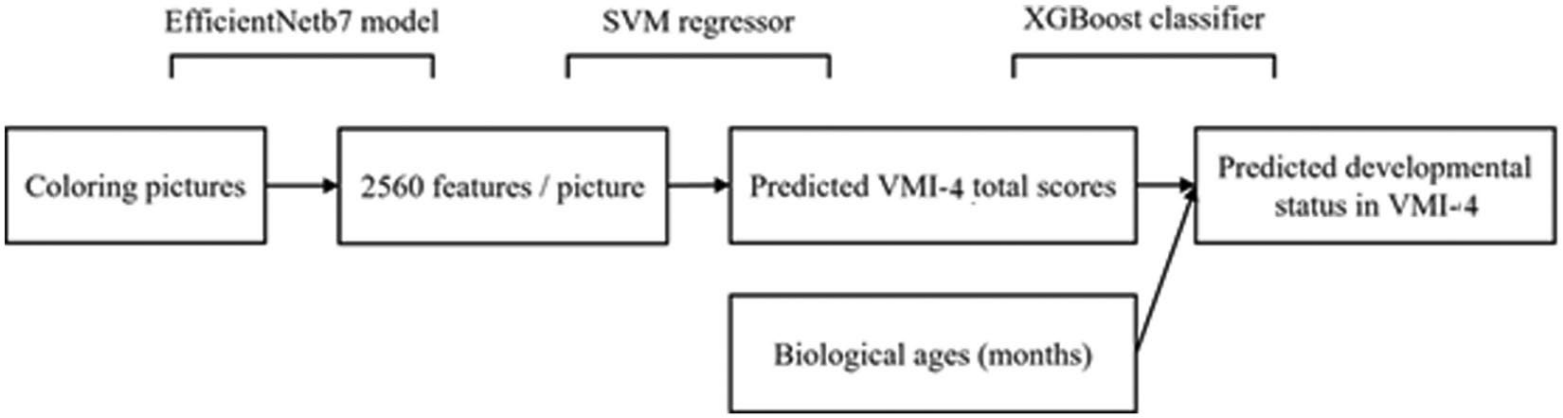
The structure of the AI model. SVM: Support Vector Machine; XGBoost: eXtreme Gradient Boosting; VMI-6: Beery–Buktenica Developmental Test of Visual–Motor Integration, Four edition.

Similar trends were observed in the testing dataset, where the SVM regressor and XGBoost classifier combination achieved the best performance, with sensitivity of 0.74, specificity of 0.81, F1-score of 0.81, and AUC of 0.83. The Youden’s J statistic was 0.55, indicating acceptable diagnostic power. Figure 2 presents the AI model structure of the best performance.

All model parameters for the three approaches are provided in Supplement 1.

## Discussions

This study aimed to use performance in a coloring activity with an AI application to assess children’s developmental status of visual–motor integration. The results of our study showed acceptable to medium diagnostic power. Therefore, the coloring activity, a fun and attractive activity for children, can be used as a screening tool for visual–motor integration and has great potential for assessing children’s development.

For the testing data, the accuracy of the AI model was 80% and the value of J was 0.554. This result was reasonable because studies have reported that visual–motor integration is required when executing coloring activities [3]. Moreover, our study used machine learning/deep learning with 5-fold cross-validation to train the model for visual–motor integration. This method made the model more robust because different samples were repeatedly tested, and similar results were obtained in our study.

SMOTE was employed to address the imbalance in the training dataset and reduce the likelihood of the AI model misclassifying children’s developmental status on the VMI-4 assessment [11]. In our study sample, as in reality, children with typical development in visual–motor integration were the majority. This high proportion could lead the AI model to predominantly predict “typical development,” yielding high accuracy but failing to effectively identify children with developmental delay. Identifying developmental delay is critical to providing timely and appropriate interventions. To tackle this issue, we applied SMOTE to the training dataset, increasing the number of children with developmental delay and balancing the data for children with and without developmental delay in visual–motor integration. However, SMOTE has two primary limitations. First, if the minority class contains noisy or mislabeled data, SMOTE may generate synthetic samples based on this noise, potentially degrading the model’s performance [18]. Second, SMOTE assumes that the minority class possesses distinct characteristics that differ significantly from the majority class, which may not always reflect real-world data. To mitigate these concerns, we used a cross-validation approach to monitor potential overfitting. The cross-validation was conducted using three approaches. First, we performed 5-fold cross-validation to identify the optimal parameters, allowing the training process to detect overfitting. Second, after tuning the hyperparameters, we applied the selected model to data without SMOTE to assess its performance on real data. Third, we tested the model on a completely new dataset to validate the performance of the trained model. The new dataset contained only real data. Since the final model testing achieved acceptable results, we may conclude that SMOTE was applicable in our case. Furthermore, the sensitivity of the AI model improved after using SMOTE, enhancing its ability to identify children’s developmental status in visual–motor integration based on their performance on a coloring activity. This upsampling technique shows promise in the medical field, where imbalanced datasets are common due to the relatively low prevalence of certain diseases or diagnoses.

Based on the manual instructions and clinical application in Taiwan, VMI-4 scores can be categorized into three subgroups: normal, at risk of developmental delay, and developmental delay. However, we did not further categorize VMI-4 scores for two reasons. First, the sample sizes for both the developmental delay and at-risk groups were already small, especially the at-risk group. Further dividing the sample into three groups would require incorporating more simulated data to maintain balance among the groups, which could compromise the authenticity of the real data. Second, we designed this machine learning model as a screening tool. The primary goal of a screening tool is to identify children at risk of developmental delay rather than to further classify them into multiple categories. Therefore, we combined the at-risk and developmental delay groups which may need more medical resources and focused on distinguishing them from the normal group. However, upon further examination of the error patterns (Supplement 2), we found a relatively high error rate in the at-risk group. This suggests that future studies should aim to increase the sample sizes of both the at-risk and developmental delay groups to improve identification accuracy.

The children recruited for our study were aged 3 to 6 years. This age range was selected because coloring is a common and popular activity among preschool children and corresponds to a period of rapid development in visual–motor integration. Decker [19] investigated the growth and decline of visual–motor abilities using the Bender Visual–Motor Gestalt Test, Second Edition. Their findings indicated that visual–motor abilities undergo rapid maturation during early childhood and continued development into middle adolescence, followed by a steady decline through adulthood and a more pronounced decline in later years. Several studies have identified the relationship between coloring activities and visual–motor integration. Additionally, Huang [1] quantified coloring ability and examined the developmental stages of coloring. They identified three stages: immature, transitional, and mature, corresponding to children aged 3 to 4 years, 4 to 5 years, and 6 years and older, respectively. These findings highlight the connection between coloring activities and visual–motor integration, making the coloring task a suitable tool for assessing visual–motor integration in children aged 3 to 6 years.

**Figure 3.**
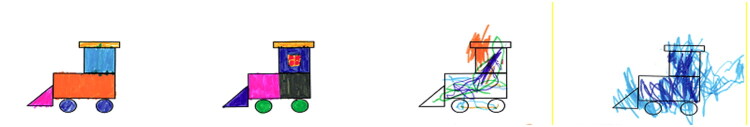
Examples of images with the highest and lowest VMI scores.

In our study, the final model integrated EfficientNetB7, an SVM regressor, and an XGBoost classifier. It is noted that the interpretability of EfficientNetB7 and the SVM regressor was relatively low. EfficientNetB7, as a deep learning convolutional neural network, inherently poses challenges in model interpretability, as it relies on automatically learned features for analysis. Deciphering the meaning of its 2,560 extracted features or identifying which image regions contribute to these feature values was particularly difficult and also beyond the scope of this study. Similarly, when these 2,560 features were input into the SVM regressor, their interpretability remained limited. To enhance understanding, we present examples of images with the highest and lowest VMI scores to help professionals better relate the image characteristics to VMI scores (Figure 3). Moreover, for the XGBoost classifier, only two variables—predicted VMI scores and children’s age—were used to classify children with and without developmental delay in visual–motor integration. Since both variables were essential for identifying developmental status from a clinical perspective, we did not further analyze their feature importance within the classifier model.

The use of AI models to examine children’s play performance so as to identify children’s visual–motor integration is an innovative approach in the pediatric rehabilitation field. This approach would increase the accessibility to testing for the early identification of children who need medical resources, especially in areas that lack professionals. However, some cautions should be noted when applying this approach. First, the AI model was trained mainly based on a train image. Different types of coloring tasks in this stage may not be applicable to this AI model, or the prediction error may be greatly increased by their use. Further studies should employ other types of coloring tasks, such as by incorporating different shapes or pens with different textures, to increase the generalizability of the results. Second, the children’s VMI scores were derived from the Taiwanese norm. It is noted that significant differences in VMI performance between eastern and western children have been found. As a result, the AI model may be more applicable to the Taiwan area or Chinese cultures.

It is essential to note the importance of applying EfficientNetB7 to actual VMI drawings by children to determine which models are more predictive of developmental delay. However, we chose the coloring task for this study for several key reasons, which we believe make it a more meaningful and practical approach. First, we aimed to create a task that is more engaging and meaningful for children. Instead of asking them to perform structured and somewhat abstract tasks like drawing a circle, square, or triangle, we incorporated a coloring activity. Coloring in a train, for example, is an activity that children can relate to and enjoy. This makes the task feel less like an assessment and more like play, which can foster better cooperation and engagement. Additionally, the coloring task is versatile and can be conducted in various settings, such as at home, in schools, or in clinics, making it more broadly applicable than traditional VMI assessments, which typically require administration by clinicians. Second, we see the coloring task as a scalable and practical tool for different contexts. Once we establish the analytical processes for the task, it can be expanded to include multiple images and not be limited to the train design. Our research team has already developed a step-by-step instructional guide that parents and teachers can easily follow. This accessibility means that caregivers, even in rural areas with limited access to medical resources, can implement the task using images of their choice. This broader applicability allows for early identification of developmental issues in areas where professional assessments might not be readily available. Finally, this study represents the initial step in our larger research project. The coloring task serves as a foundational tool for analyzing both visual–motor integration and fine motor skills. Unlike traditional VMI assessments, this task has the potential to provide insights into multiple developmental domains simultaneously. For example, coloring activities could also reflect cognitive and fine motor abilities. Our ultimate goal is to develop an assessment approach that identifies various developmental areas in a more comprehensive and efficient way. By building upon the results of this study, we aim to create a task that can not only be used to evaluate developmental status but also serve as a valuable tool for parents, teachers, and clinicians alike.

Based on the results of our study, an app or cloud platform could be developed. Specifically, the machine learning model could be embedded in an app or webpage. Users such as clinicians, school teachers, or caregivers could let children color the train using the template provided in this paper. After children completed the task, adults could scan their products, upload a coloring picture to the webpage or an app, and provide the children’s age. The app or the webpage could then process this information, and the machine learning models could proceed to create an output indicating whether the children have visual–motor integration problems or not. This method could greatly increase the accessibility of the evaluation and may be an efficient screening tool for VMI. However, in this study, only pictures of a train were used to train the machine learning model. Therefore, the pictures used for coloring should be restricted to the pictures used in our study. Future studies are warranted to further use other coloring pictures in the machine learning model to increase its generalizability.

## Limitations and future work

Our study had two limitations. The first is that group activities rather than individual activities were used to collect children’s coloring products. Children’s performance may be influenced by the assessment environment (e.g. peers’ movements and noise may distract them). Thus, children’s performance in group activities may differ from that in individual activities. More studies are needed to collect children’s activity products in individual activities to compare the differences between the two data collection methods. Second, the children were recruited from kindergartens; therefore, most of the children in this study had typical development. Future studies should recruit children in rehabilitation clinics to increase the sample variation and further increase the generalizability of the AI model.

## Conclusions

Our study aimed to investigate whether coloring, a popular activity for children, can be used as a screening tool for visual–motor integration by adopting AI techniques. Our results showed that the trained AI model had acceptable-to-medium diagnostic power. The results indicate that the trained AI model has great potential as an evaluation tool for predicting children’s developmental status of visual–motor integration.

## Supplementary Material

Supplement 2.docx

Supplement 1.docx

## Data Availability

The data that support the findings of this study are available from the corresponding author, [CYH], upon reasonable request.
