# Supplementary material for: Using a coloring activity to identify children’s development of visual–motor integration: an application of artificial intelligence
Source: Ann Med. 2025 Nov 3;57(1):2578725. doi: 10.1080/07853890.2025.2578725 (PMC12584823; doi:10.1080/07853890.2025.2578725)
Supplement: Supplement 2.docx [file IANN_A_2578725_SM9931.docx]

Supplement 2: The identification error of the training and testing datasets

| ID | Age | VMI score | Predicted VMI score | Developmental status | Predicted developmental status | Picture |
| --- | --- | --- | --- | --- | --- | --- |
| Training dataset | | | | | | |
| DG(B)016 | 5y2m | 10 | 13 | Suspect delayed* | Normal | 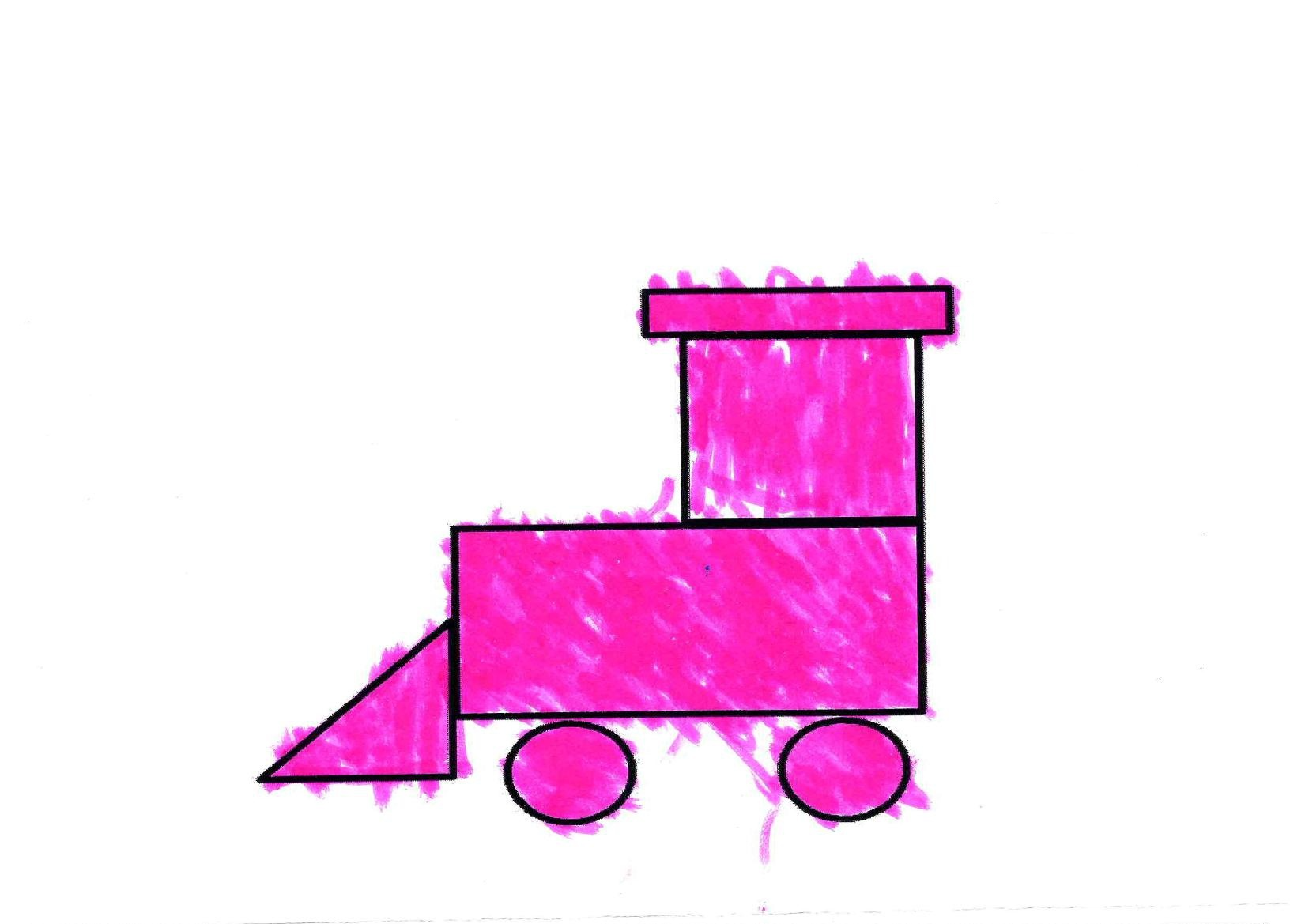 |
| MY(B)018 | 5y7m | 12 | 10 | Suspect delayed | Normal | 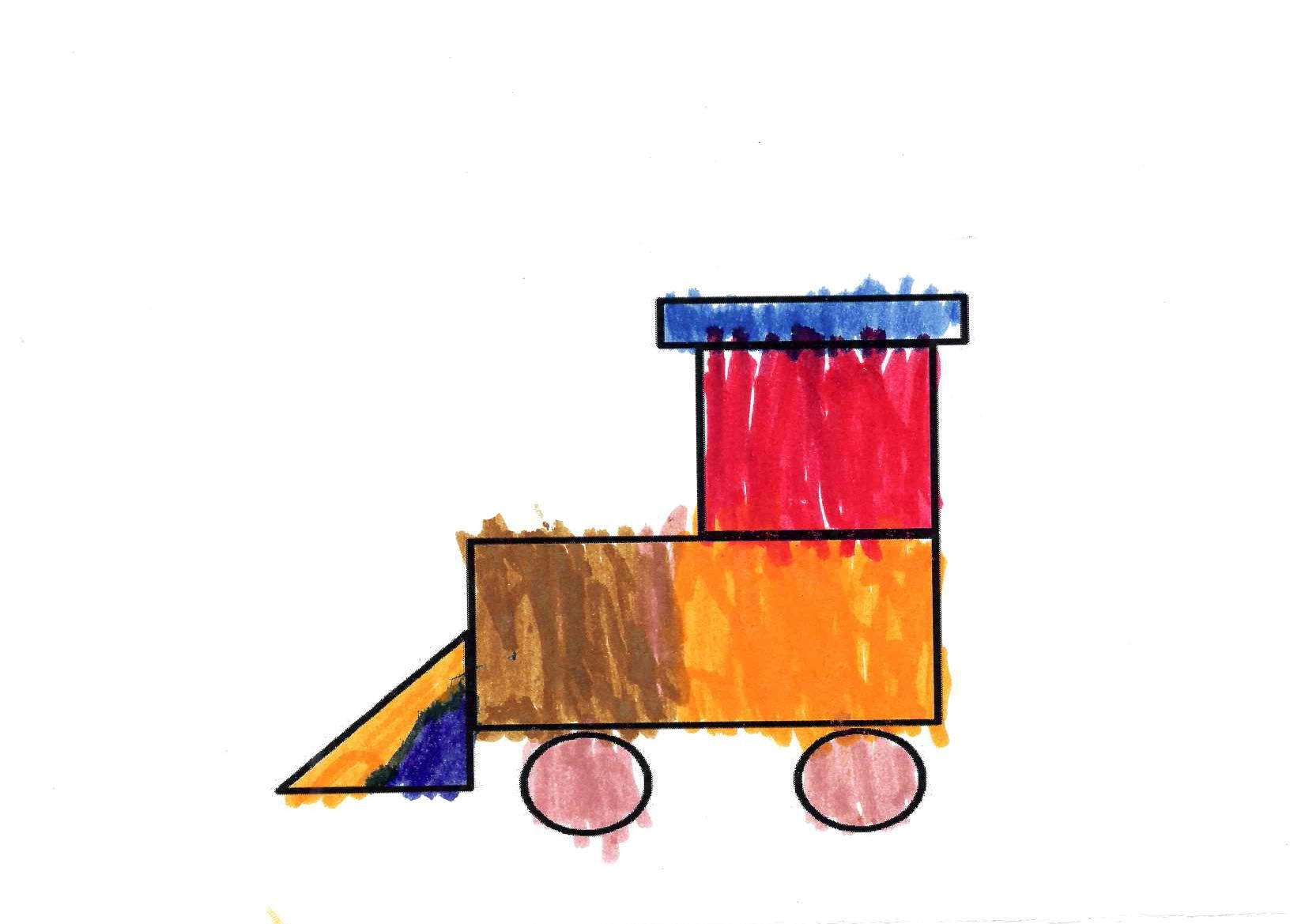 |
| MY(B)114 | 4y3m | 7 | 3 | Suspect delayed | Normal | 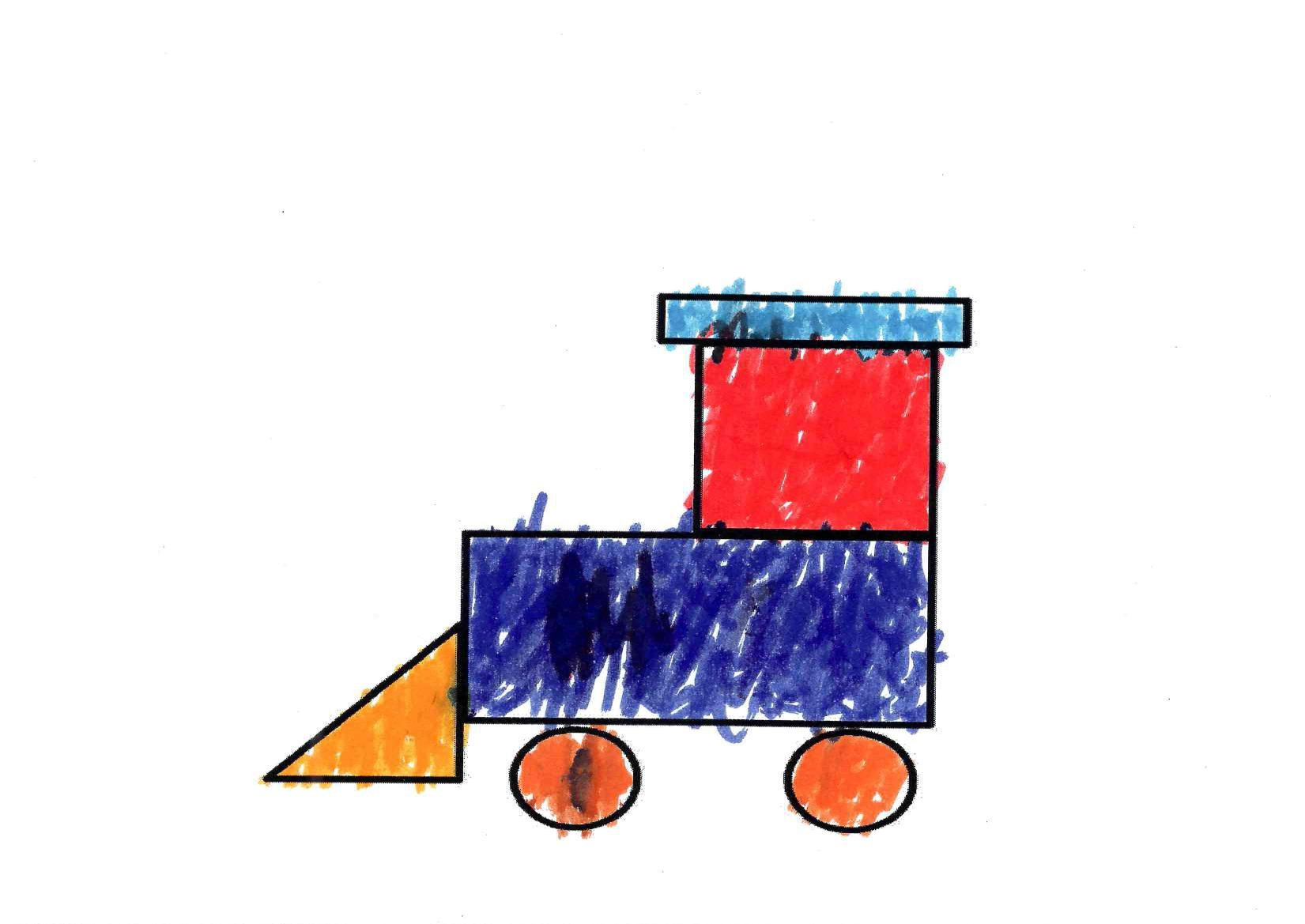 |
| MY(B)156 | 4y6m | 7 | 10 | Suspect delayed | Normal | 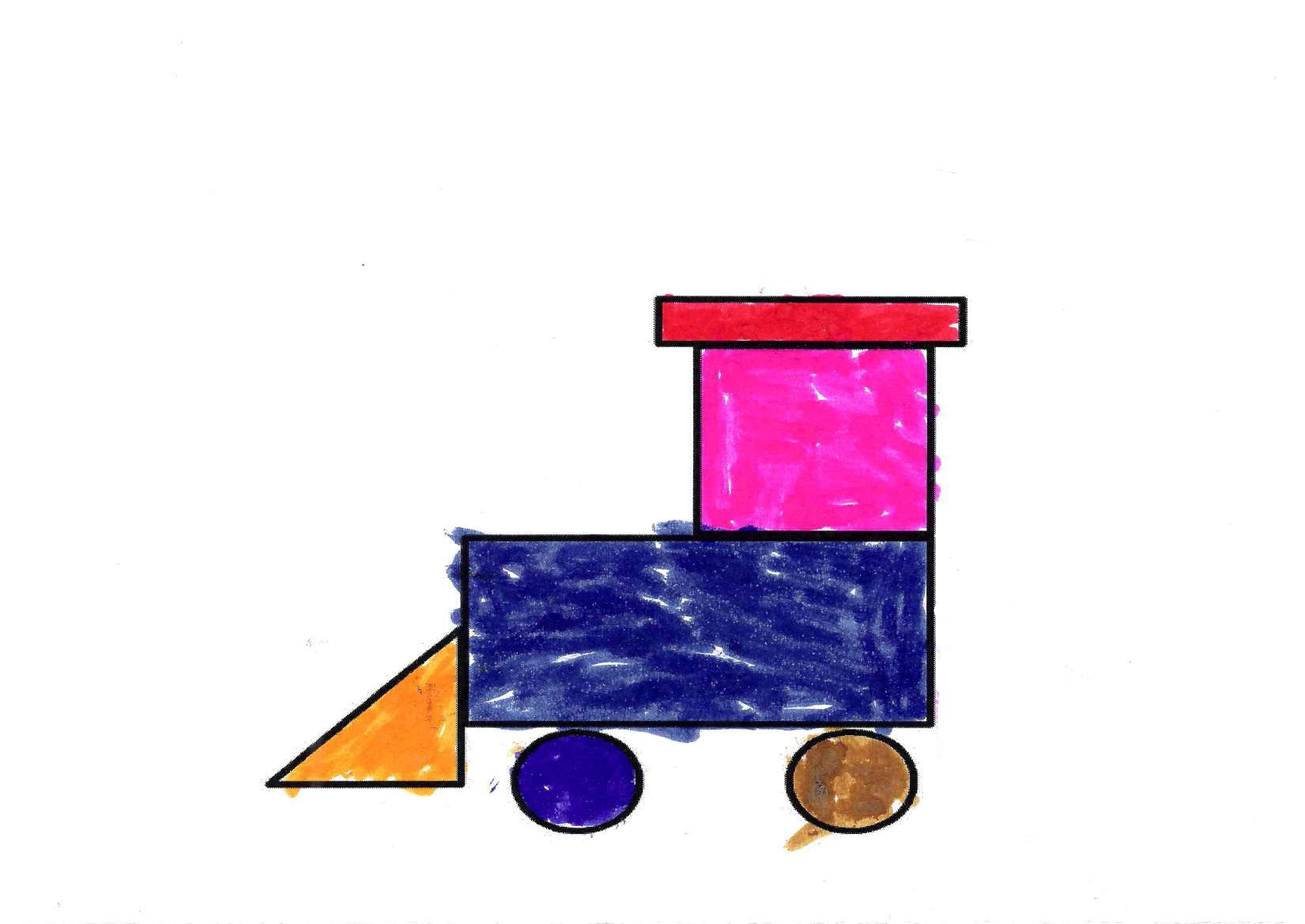 |
| MY(B)203 | 4y8m | 9 | 5 | Suspect delayed | Normal | 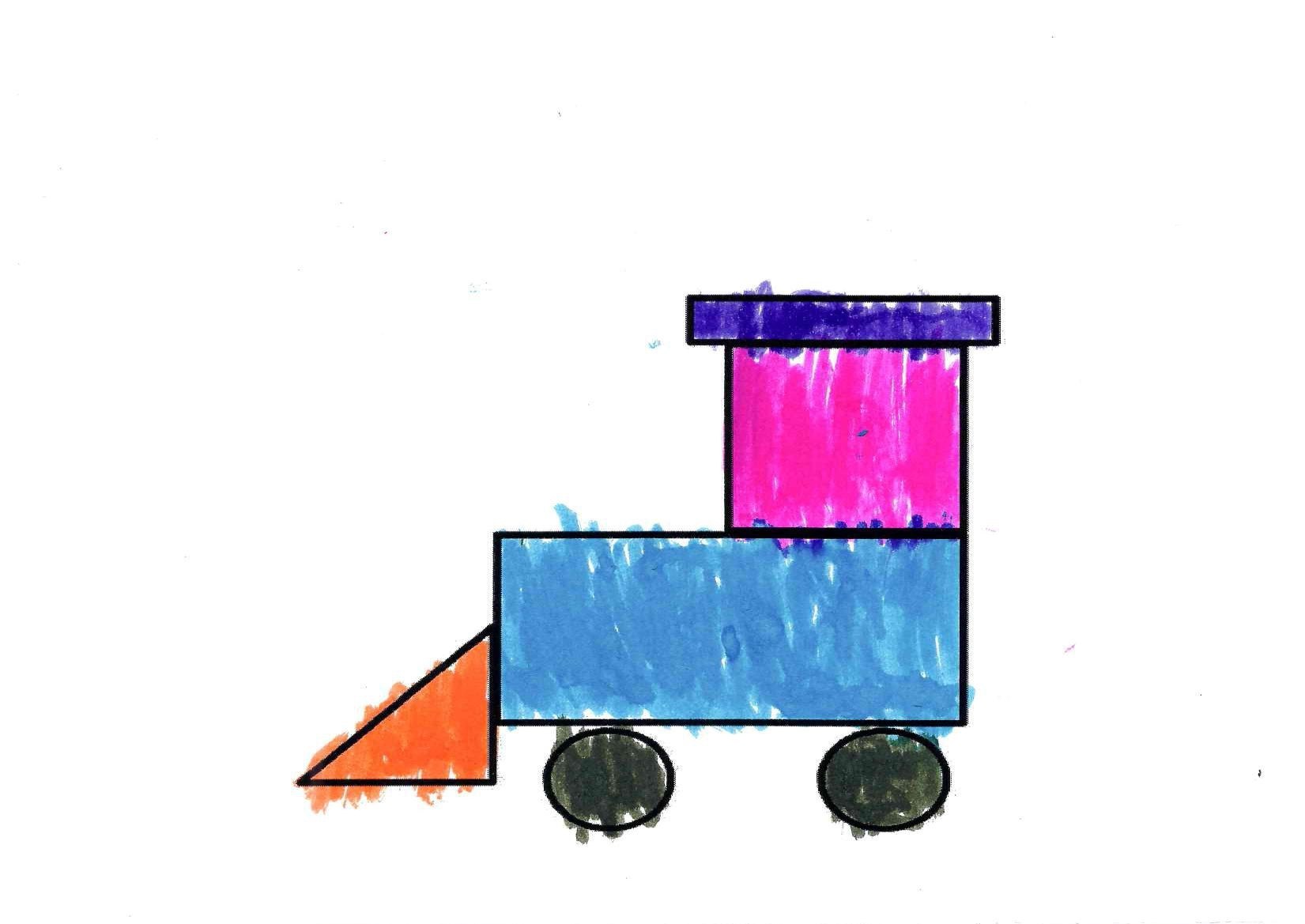 |
| Testing dataset | | | | | | |
| DG(B)003 | 6y3m | 20 | 15 | Normal | Delayed | 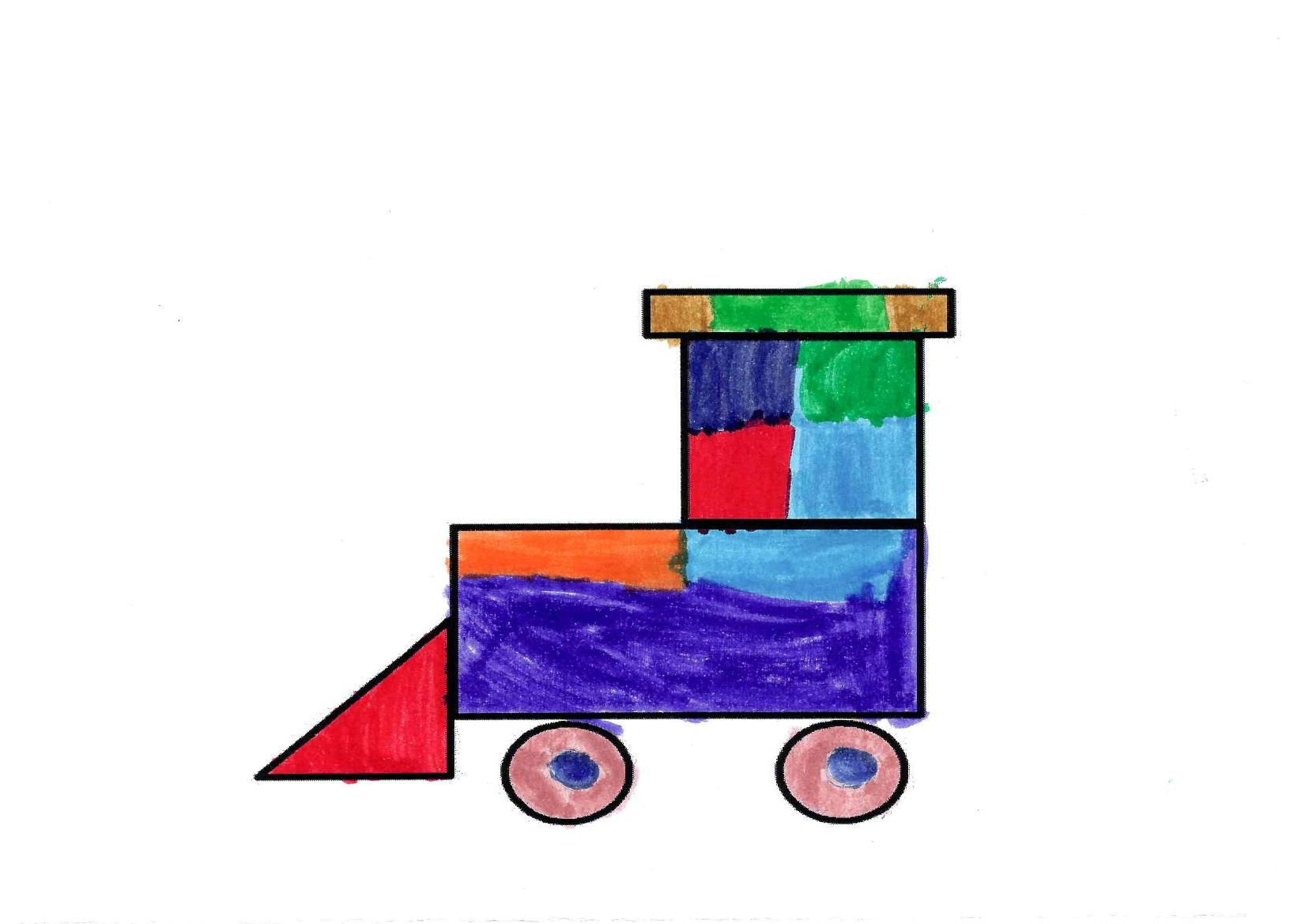 |
| DT(B)037 | 5y11m | 14 | 12 | Normal | Delayed | 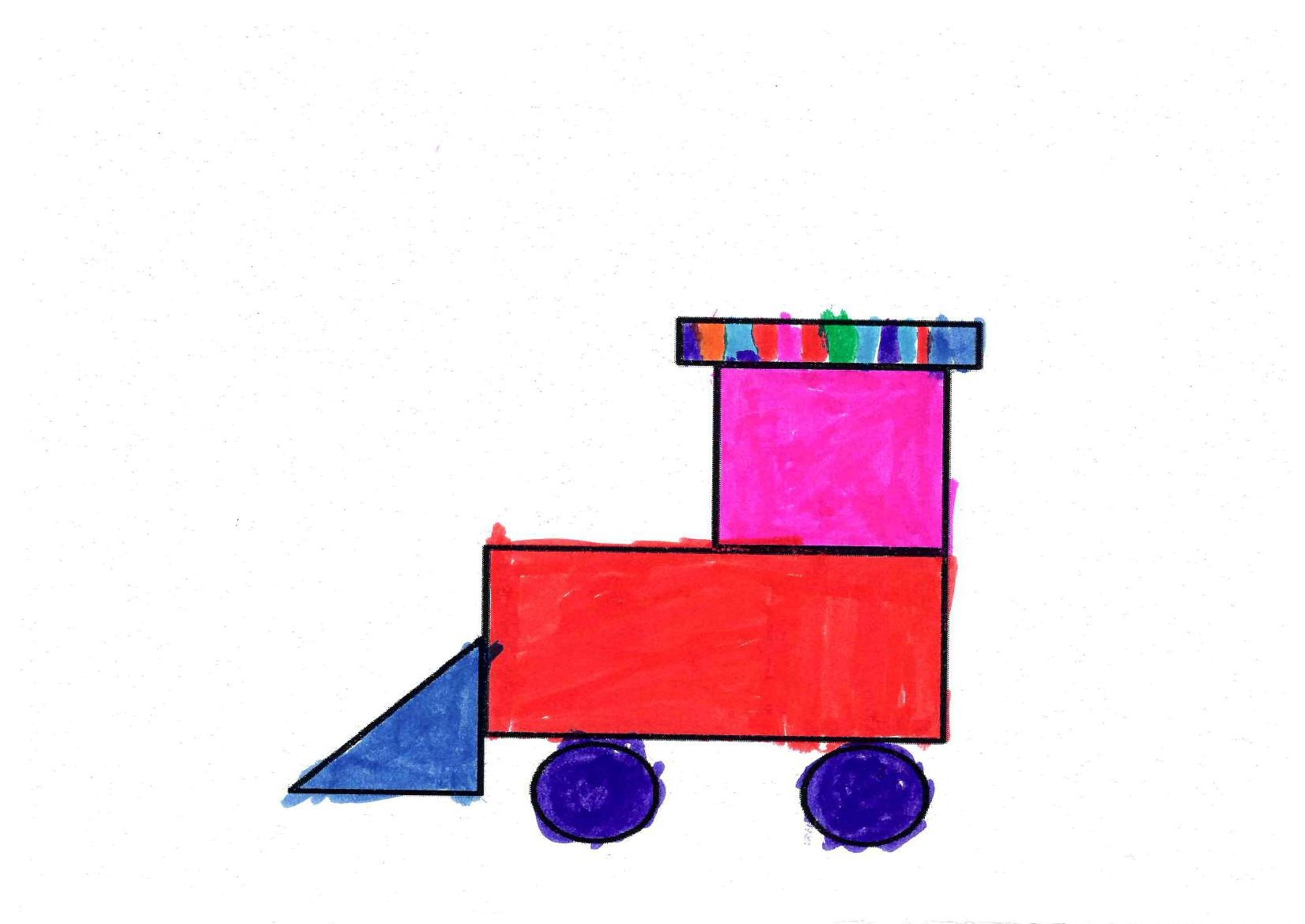 |
| DSO(B)014 | 5y7m | 15 | 12 | Normal | Delayed | 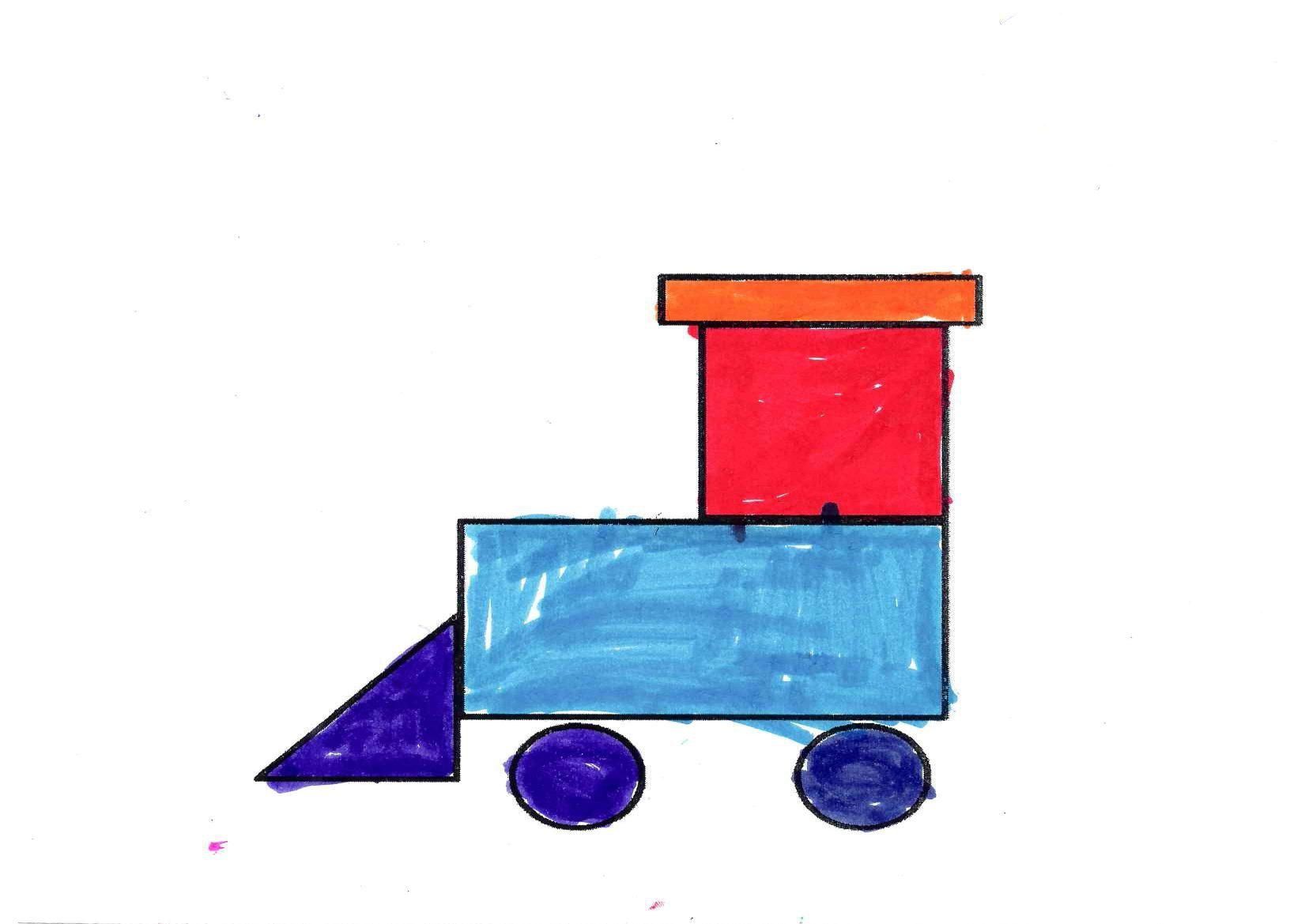 |
| MY(B)003 | 6y2m | 15 | 10 | Normal | Delayed | 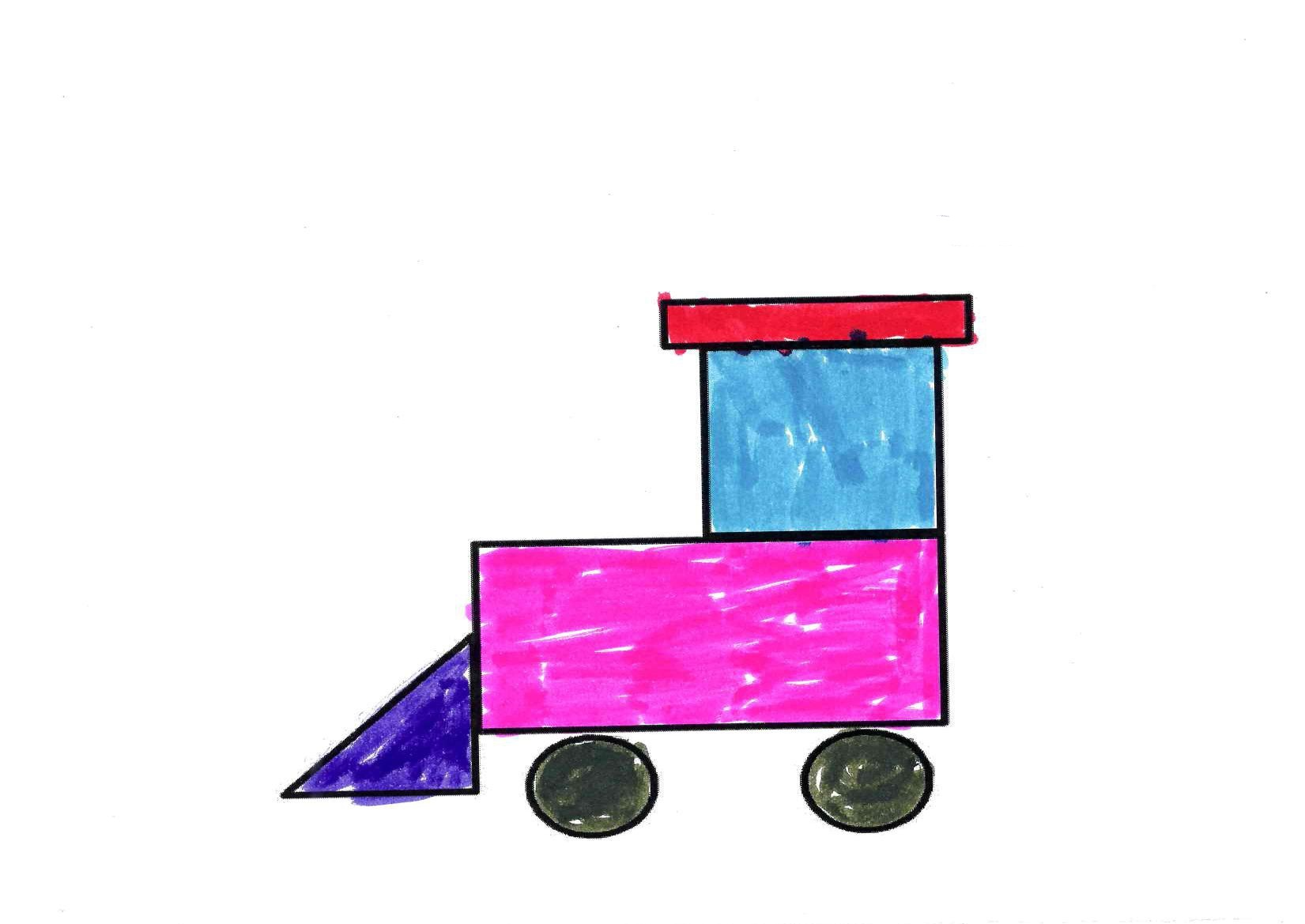 |
| MY(B)070 | 6y3m | 18 | 17 | Normal | Delayed | 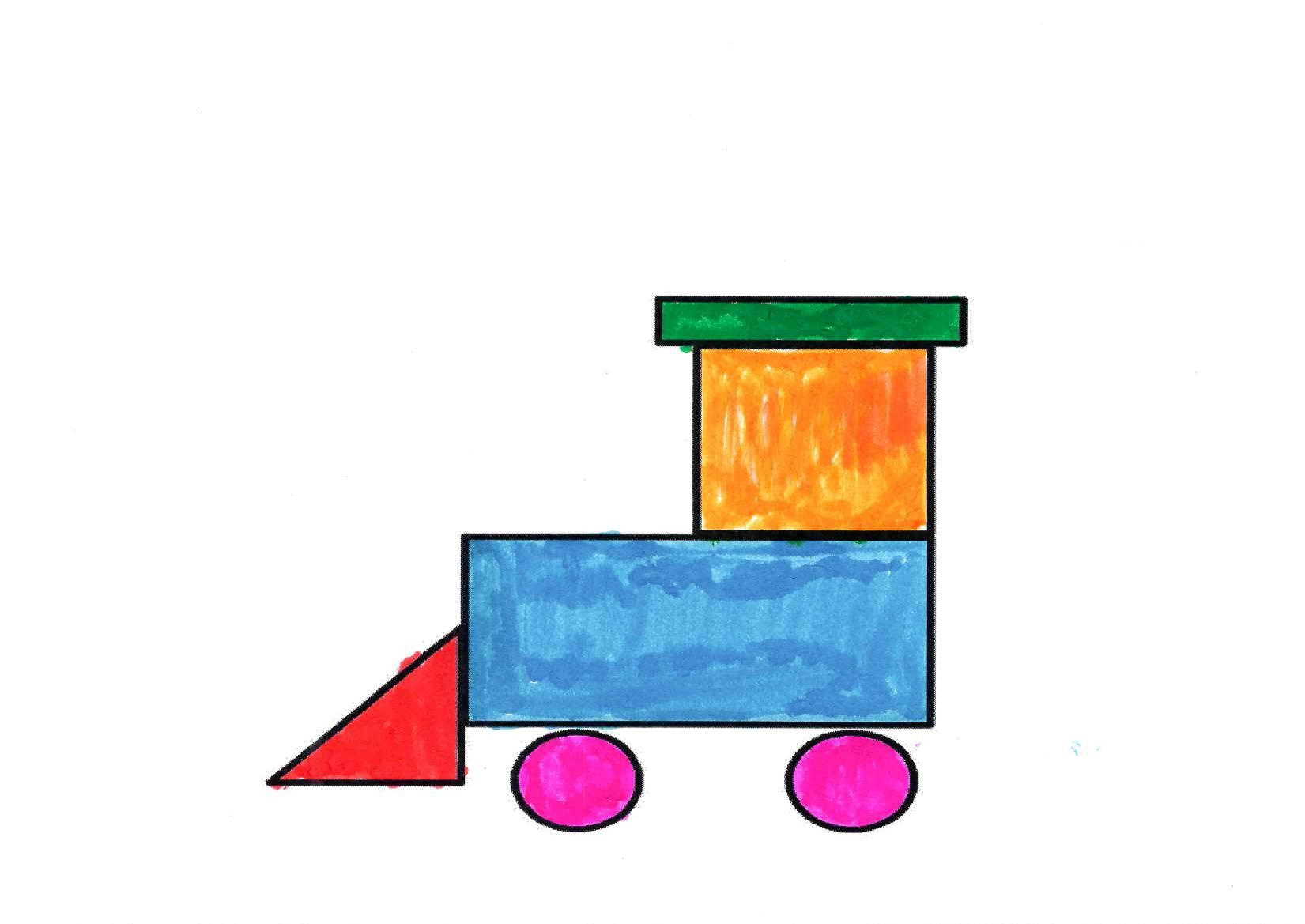 |
| NT(B)024 | 5y8m | 17 | 16 | Normal | Delayed | 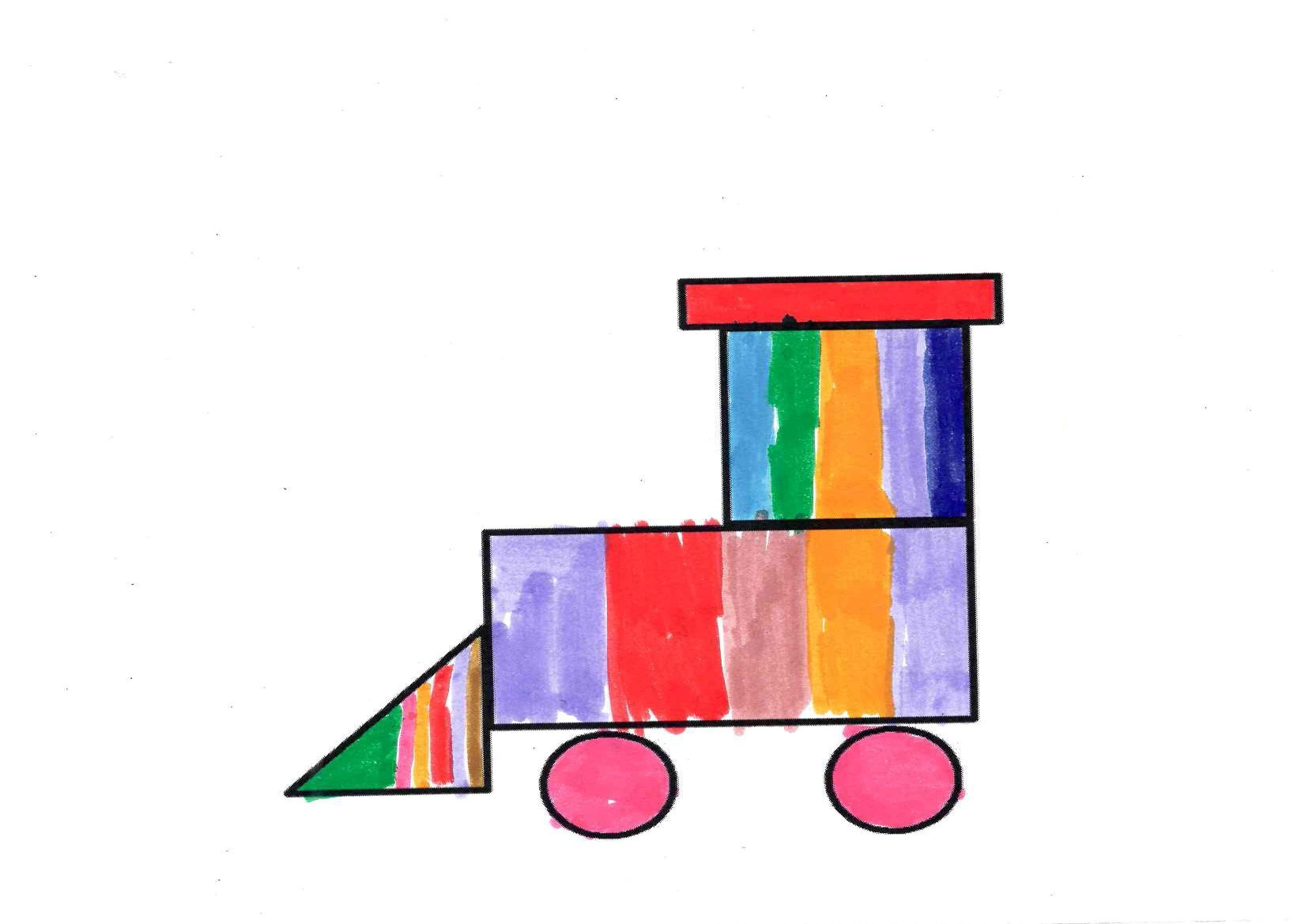 |
| YC(B)002 | 5y0m | 12 | 15 | Normal | Delayed | 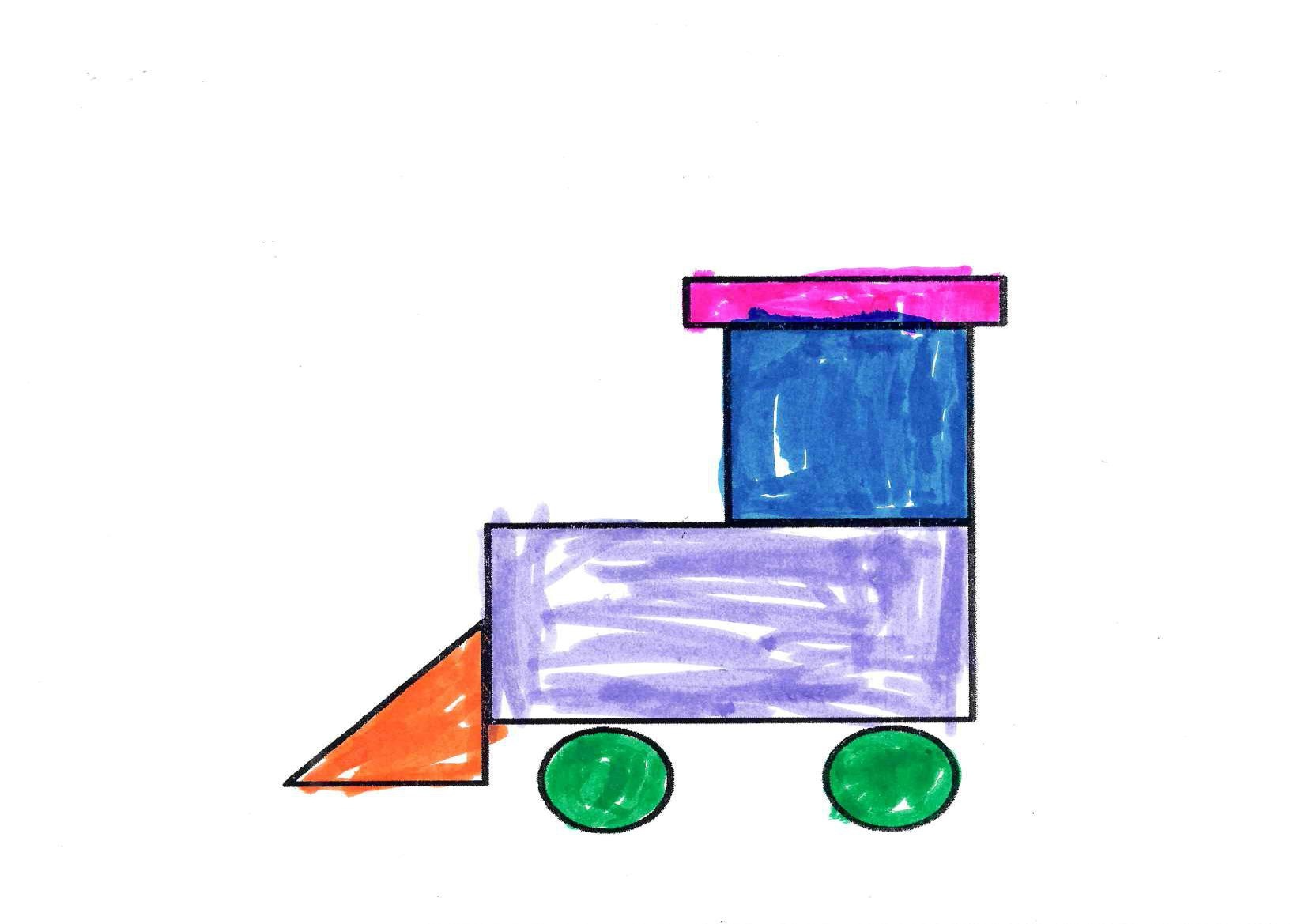 |
| DG(B)043 | 4y7m | 7 | 9 | Suspect delayed | Normal | 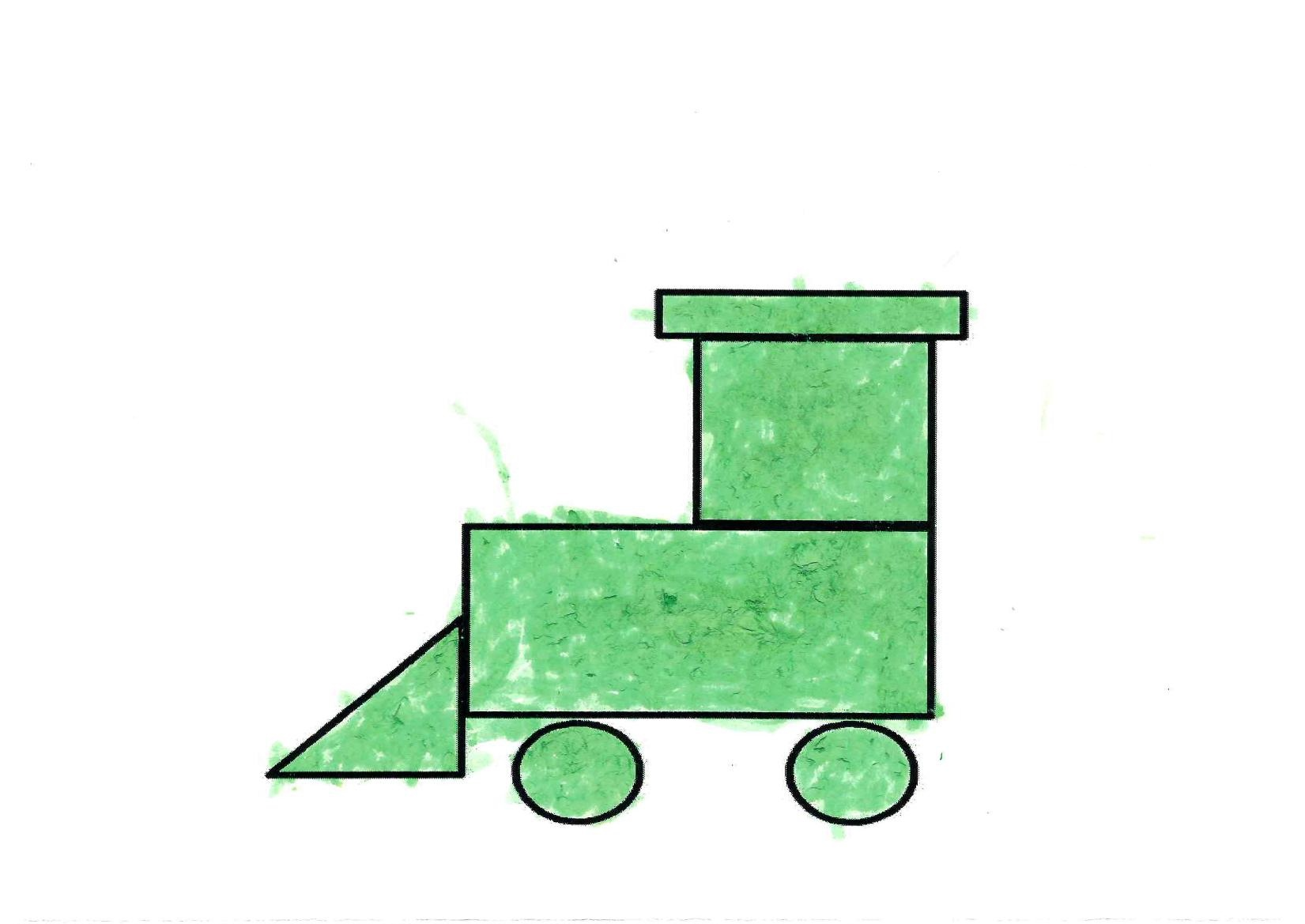 |
| DT(B)005 | 5y8m | 12 | 15 | Suspect delayed | Normal | 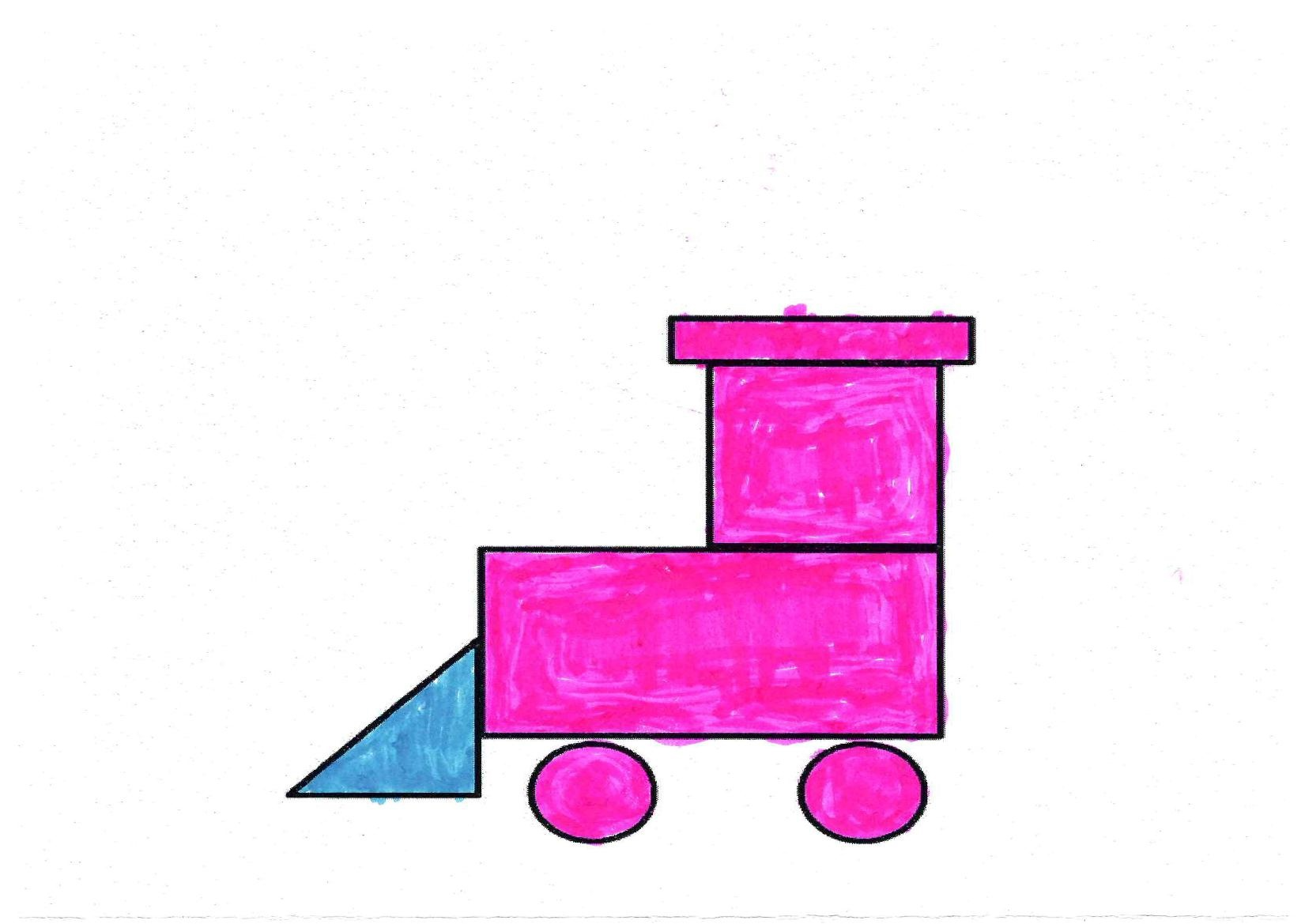 |
| DT(B)026 | 4y3m | 7 | 5 | Suspect delayed | Normal | 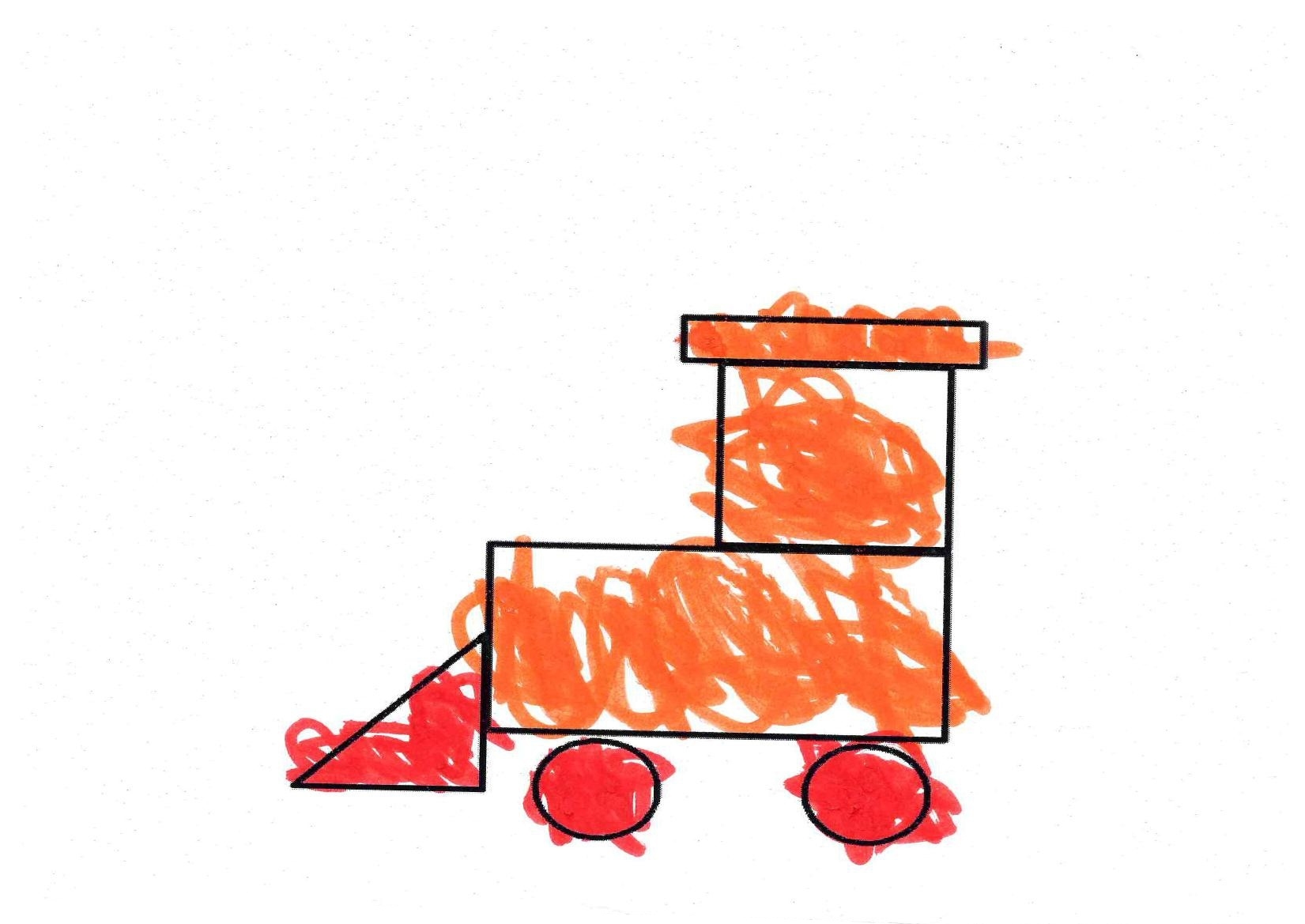 |
| MY(B)002 | 6y2m | 13 | 12 | Suspect delayed | Normal | 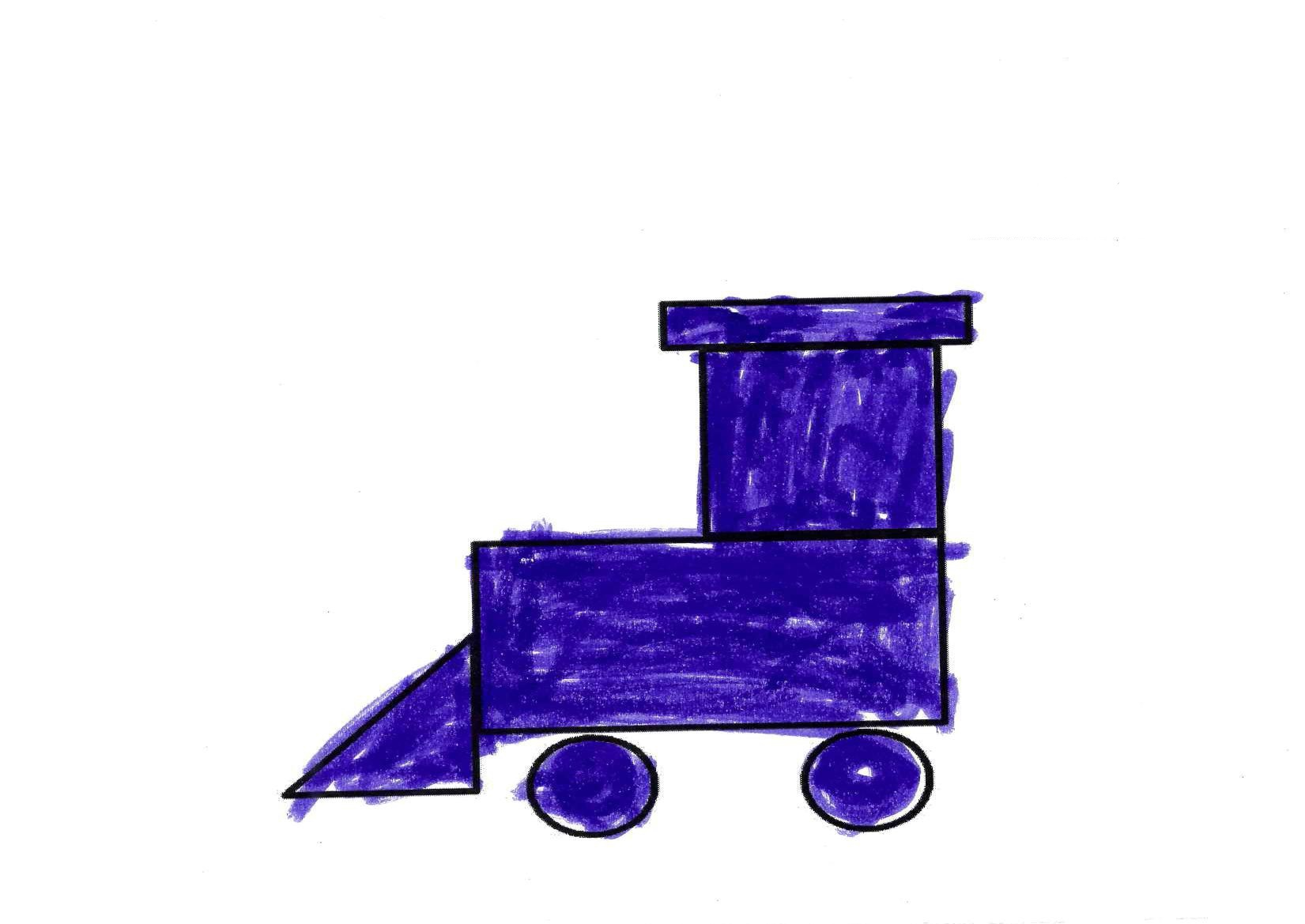 |
| MY(B)017 | 5y7m | 12 | 15 | Suspect delayed | Normal | 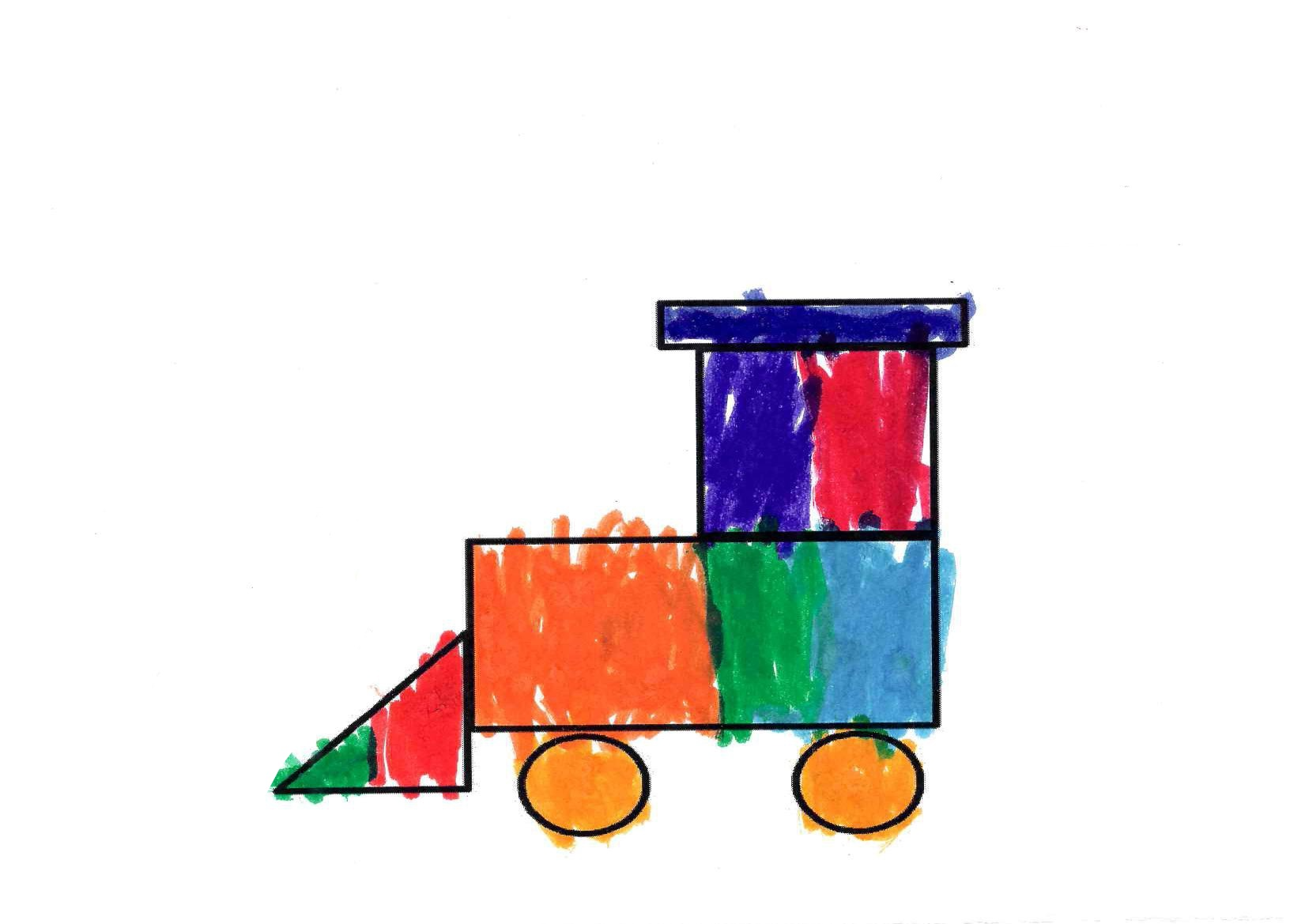 |
| MY(B)031 | 6y0m | 13 | 14 | Suspect delayed | Normal | 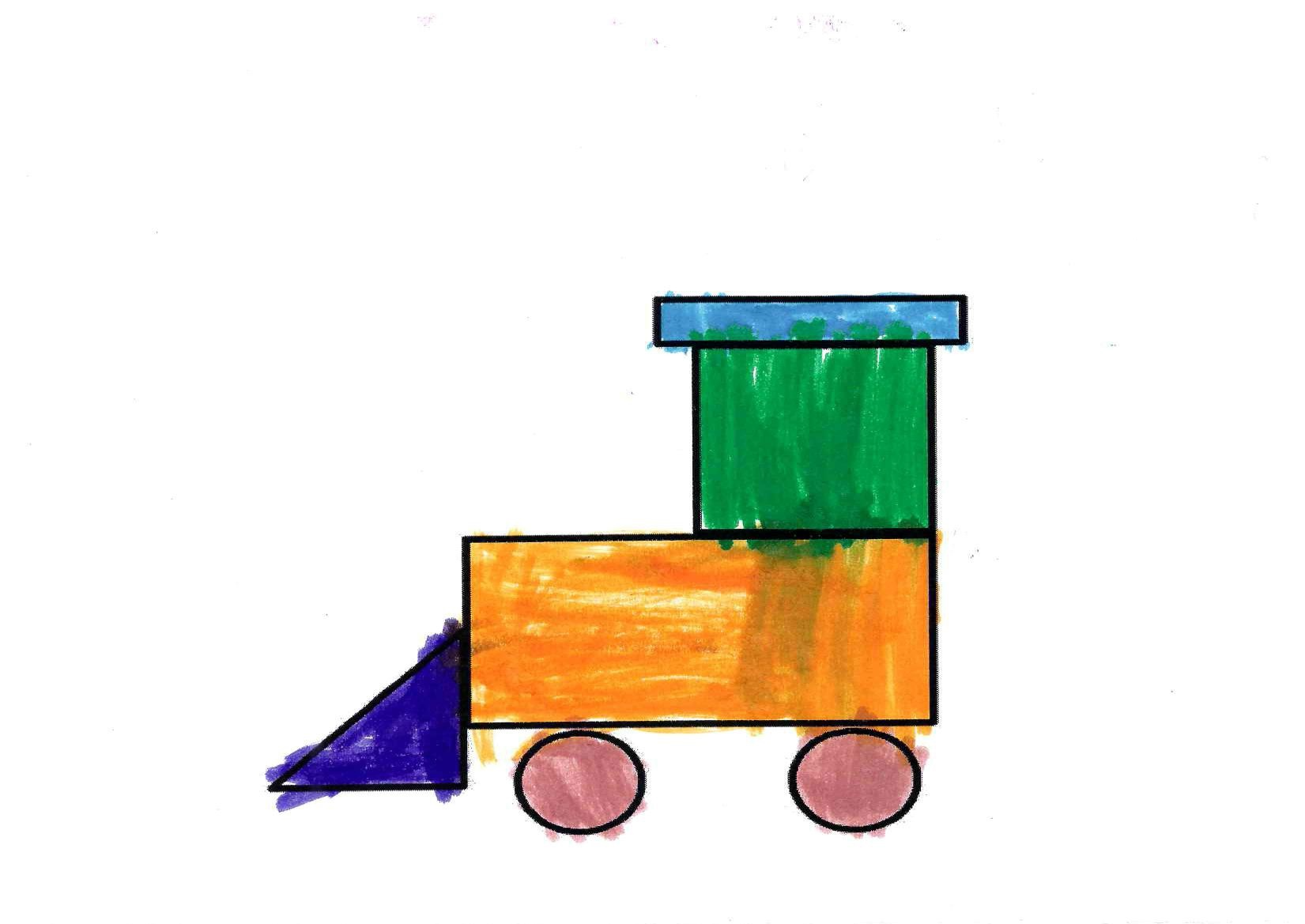 |
| NT(B)042 | 5y11m | 11 | 16 | Suspect delayed | Normal | 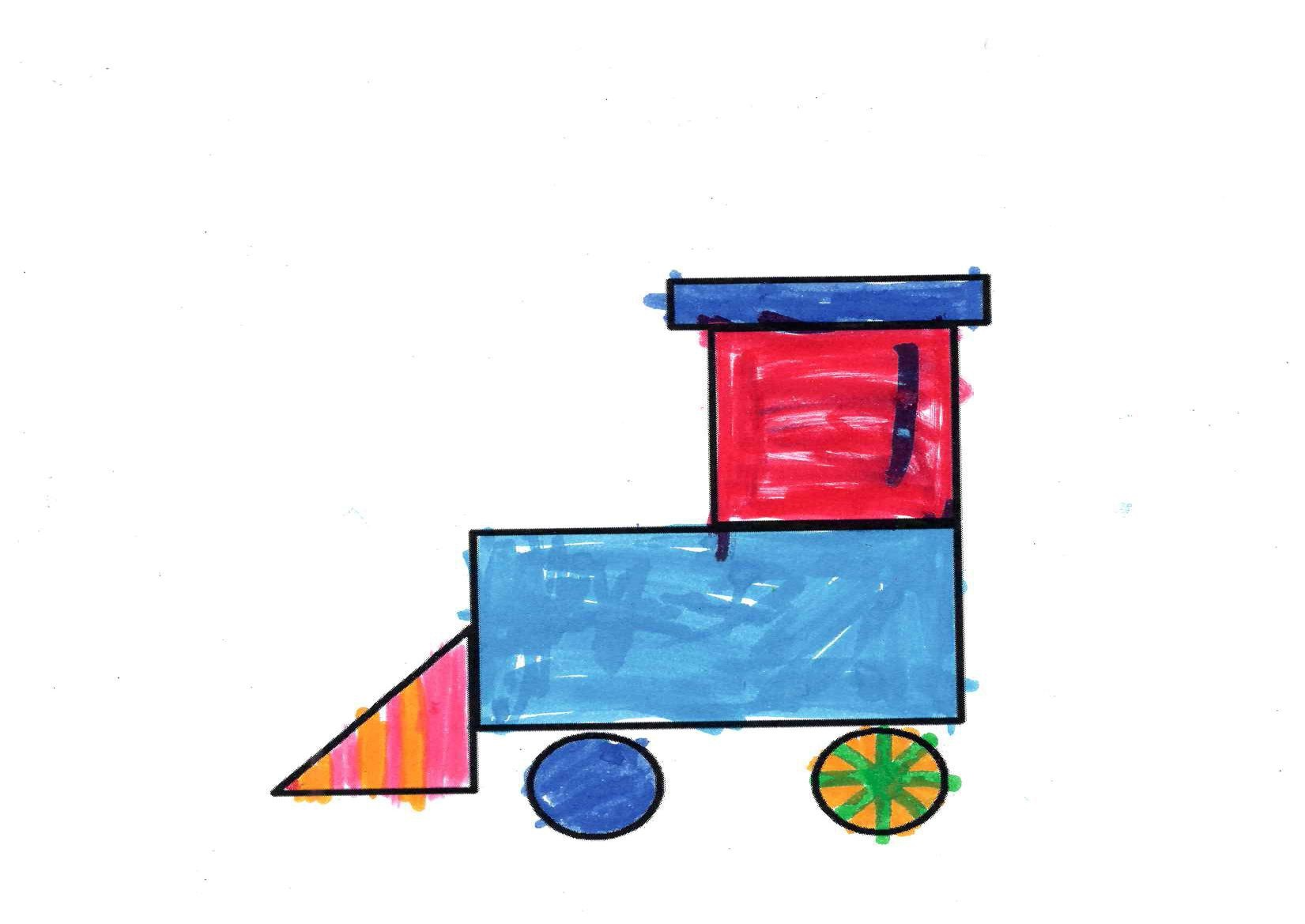 |
| NT(B)043 | 4y4m | 8 | 11 | Suspect delayed | Normal | 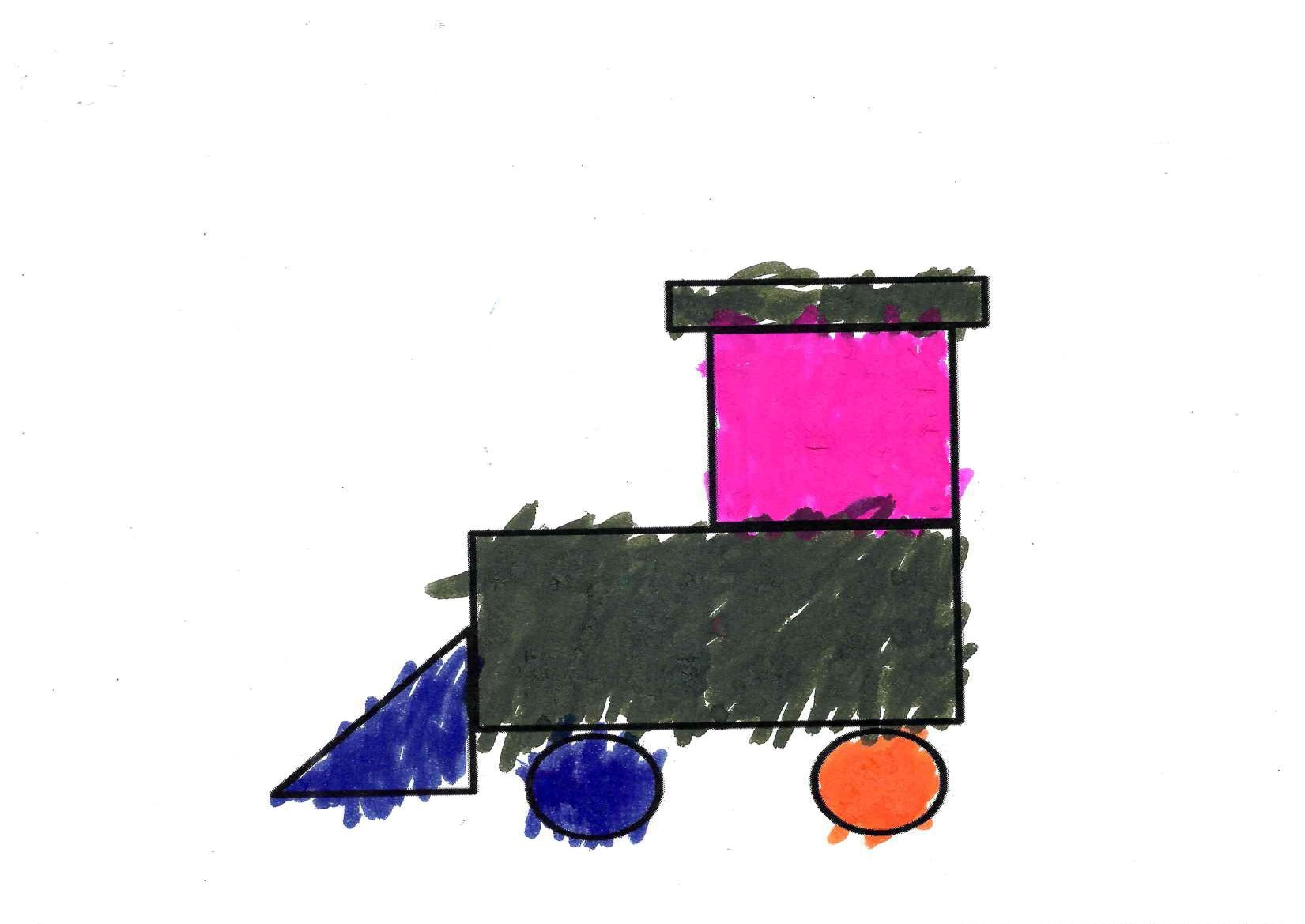 |
| MY(B)119 | 4y0m | 4 | 5 | Delayed | Normal | 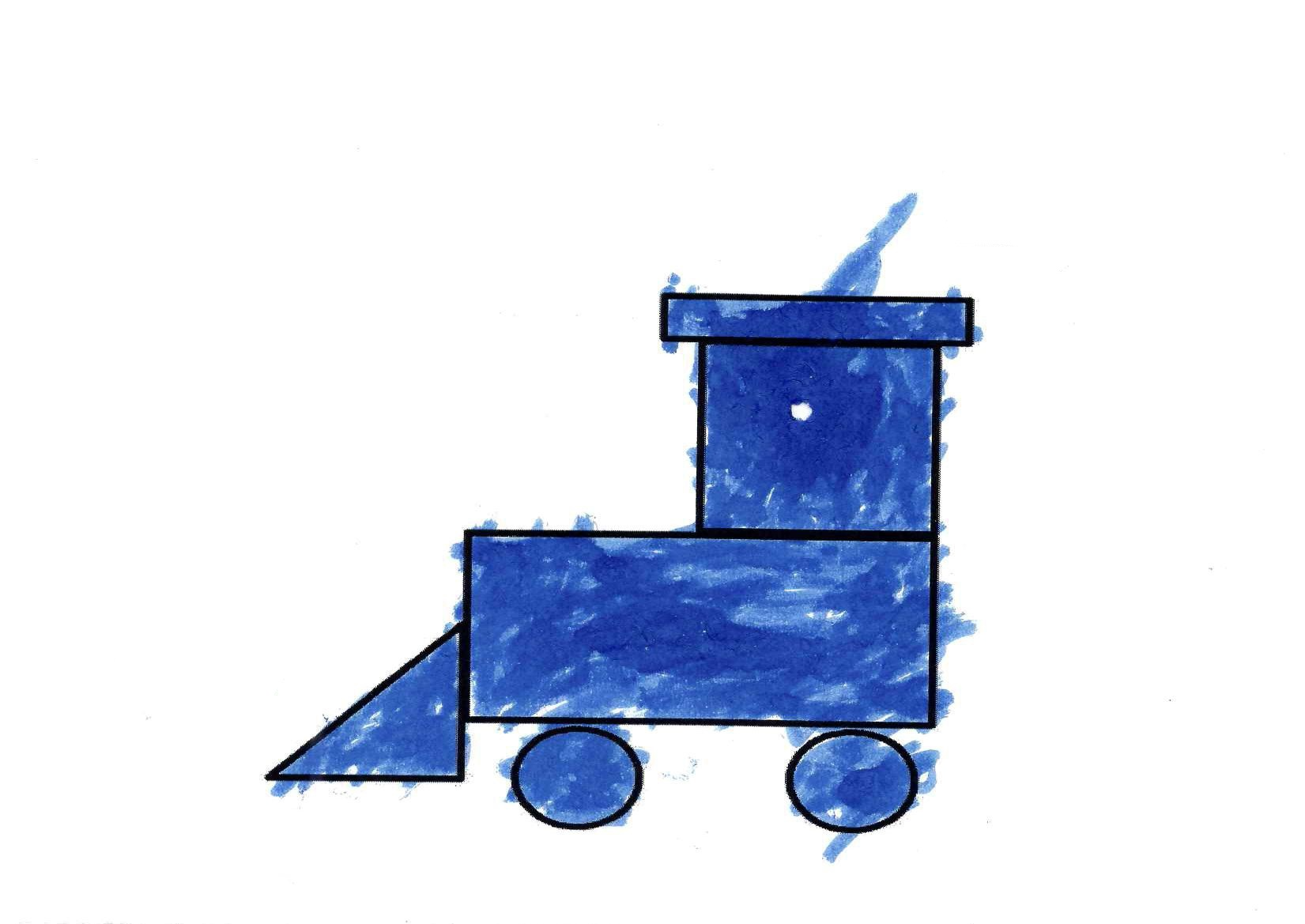 |
| DSO(B)015 | 5y6m | 7 | 6 | Delayed | Normal | 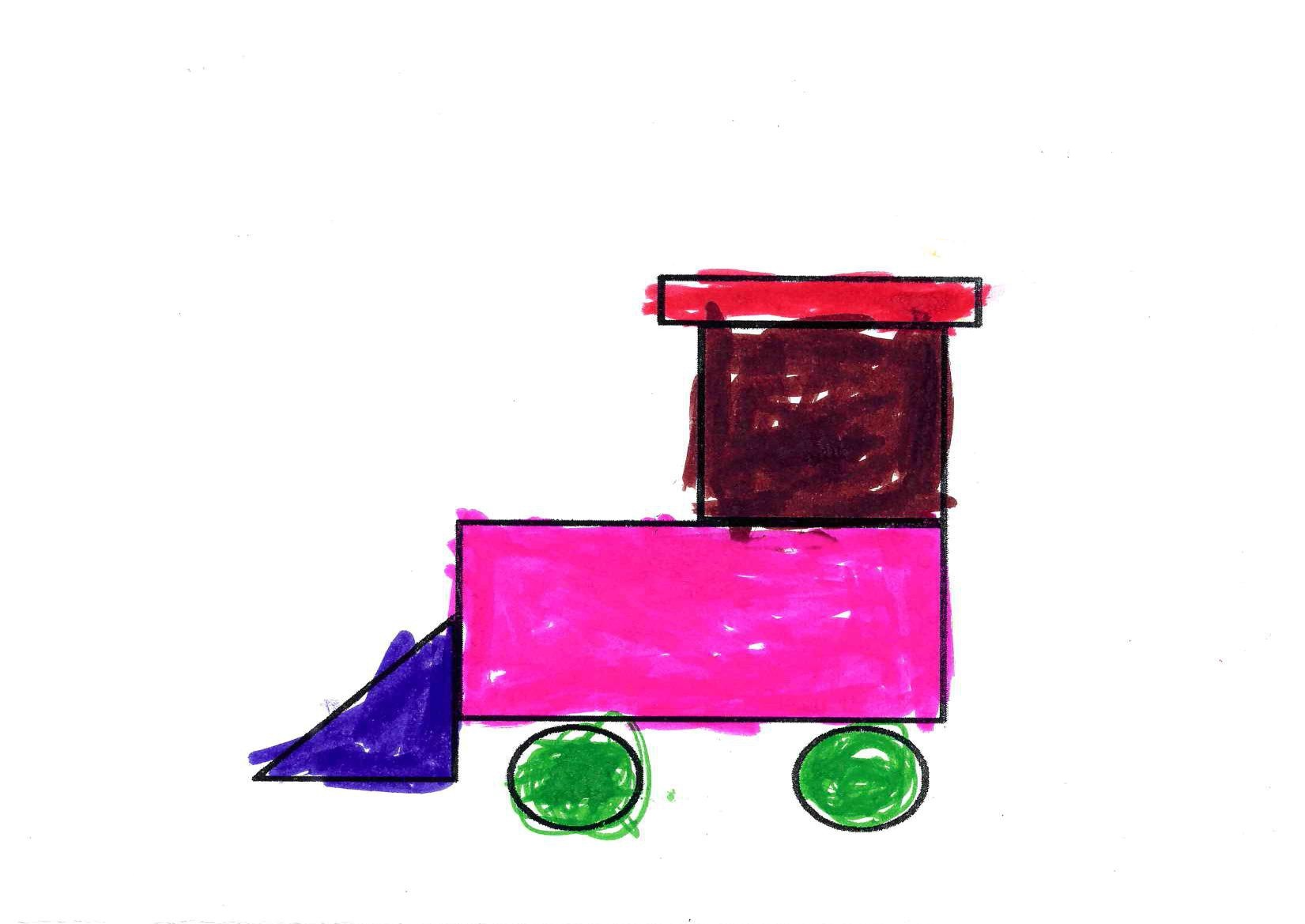 |

***** Suspected delay was recoded as ‘delayed’ in the analyses.
