# Supplementary material for: Using a coloring activity to identify children’s development of visual–motor integration: an application of artificial intelligence
Source: Ann Med. 2025 Nov 3;57(1):2578725. doi: 10.1080/07853890.2025.2578725 (PMC12584823; doi:10.1080/07853890.2025.2578725)
Supplement: Supplement 1.docx [file IANN_A_2578725_SM9930.docx]

| Supplement 1. The hyperparameters of machine learning models | | | | |
| --- | --- | --- | --- | --- |
| **Approach 1** | XGBoost Regressor | CatBoost Regressor | RF Regressor | SVM Regressor |
|  | colsample_bytree: 0.82, learning_rate: 0.11473684210526315,  max_depth: 3, min_child_weight: 1  n_estimators: 150,  subsample: 0.7842105263157895 | depth: 4, iterations: 145, learning_rate: 0.09631578947368422 | max_features: 'sqrt', n_estimators: 180, max_depth: 7,  bootstrap: False | C: 10.0, 'gamma': 0.01 |
| **Approach 2** | XGBoost Regressor | CatBoost Regressor | RF Regressor | SVM Regressor |
|  | colsample_bytree': 0.9266666666666666, 'learning_rate': 0.11473684210526315, 'max_depth': 14, 'min_child_weight': 3, 'n_estimators': 140, 'subsample': 0.7526315789473684 | depth': 6, 'iterations': 130, 'learning_rate': 0.08526315789473685 | max_features': 'sqrt', 'n_estimators': 170, 'max_depth': 7, 'bootstrap': False | C': 10.0, 'gamma': 0.01 |
| **Approach 3** | XGBoost Regressor | CatBoost Regressor | RF Regressor | SVM Regressor |
| XGBoost Classifier | colsample_bytree: 0.98, learning_rate: 1.005, max_depth: 7, min_child_weight: 3, n_estimators: 110, subsample: 0.75 | colsample_bytree: 0.98, learning_rate: 1.005, max_depth: 5, min_child_weight: 3, n_estimators: 110, subsample: 0.75 | colsample_bytree: 0.98, learning_rate: 0.5075, max_depth: 5, min_child_weight: 3, n_estimators: 140, subsample: 0.75 | colsample_bytree: 0.98, learning_rate: 0.5075, max_depth: 5, min_child_weight: 3, n_estimators: 140, subsample: 0.75 |
| CatBoost Classifier | depth: 4, iterations: 135, learning_rate: 0.07052631578947369 | depth: 8, iterations: 140, learning_rate: 0.05210526315789474 | depth: 8, iterations: 145, learning_rate: 0.06315789473684211 | depth: 8, iterations: 140, learning_rate: 0.08526315789473685 |
| RF Classifier | bootstrap: True, criterion: entropy, max_depth: 6, max_features: sqrt, n_estimators: 120 | bootstrap: True, criterion: gini, max_depth: 7, max_features: sqrt, n_estimators: 130 | bootstrap: True, criterion: entropy, max_depth: 7, max_features: log2, n_estimators: 100 | bootstrap: True, criterion: gini, max_depth: 7, max_features: log2, n_estimators: 120 |
| SVM Classifier | C: 0.5357894736842106, gamma: 0.5357894736842106 | C: 2.6389473684210527, gamma: 0.5357894736842106 | C: 4.2163157894736845, gamma: 0.5357894736842106 | C: 1.0615789473684212  gamma: 0.5357894736842106 |
